# ZFP42 maintains stemness and rhythmic transcription in human epidermal stem and progenitor cells via CRY1

**DOI:** 10.1038/s42003-026-09576-0

**Published:** 2026-01-21

**Authors:** Shuiying Gao, Hao Tan, Shuqia Xu, Zhaoyu Zhang, Yushuang Sun, Yaqiong Li, Dan Jian, Xiaowen Qi, Qing Tang, Run Chen, Dongyu Wang, Miao Zhen, Peng Wang, Bin Shu, Jingting Li

**Affiliations:** 1 Institute of Precision Medicine, The First Affiliated Hospital, Sun Yat-Sen University, Guangzhou, Guangdong China; 2 Department of Gynecology, Guangdong Provincial Clinical Research Center for Obstetrical and Gynecological Diseases, The First Affiliated Hospital, Sun Yat-Sen University, Guangzhou, Guangdong China; 3 Department of Plastic Surgery, The First Affiliated Hospital, Sun Yat-Sen University, Guangzhou, Guangdong China; 4 Department of Obstetrics and Gynecology, The First Affiliated Hospital, Sun Yat-Sen University, Guangzhou, Guangdong China; 5 Department of Burns, The First Affiliated Hospital, Sun Yat-Sen University, Guangzhou, Guangdong China

**Keywords:** Skin stem cells, Circadian rhythms

## Abstract

The role of circadian rhythm in regulating stemness in adult stem cells remains unclear. We investigated this in human epidermal stem and progenitor cells (EPSCs), finding that ~10% of expressed genes exhibit rhythmicity, with shared genes between fetal and adult EPSCs enriched in critical biological processes including the cell cycle, senescence, and apoptosis. Promoter motif analysis revealed ZFP42, a pluripotent stem cell marker, enriched in fetal rhythmic genes. ZFP42 knockdown led to the loss of stemness in human EPSCs and reduced expression of Cryptochrome Circadian Regulator 1 (CRY1), a core component of the molecular circadian clock that functions as a transcriptional repressor within the CLOCK-BMAL1 feedback loop, resulting in decreased cell proliferation and increased differentiation gene expression. These results highlight the critical role of ZFP42 in the circadian regulation of epidermal homeostasis, linking stemness maintenance to circadian mechanisms. Our findings deepen the understanding of how circadian rhythms govern epidermal stem cell functions.

## Abstract (web summary)

Circadian rhythms shape epidermal stem cell fate by regulating rhythmic gene expression in human EPSCs. ZFP42 maintains stemness by sustaining CRY1 expression, promoting proliferation and restraining differentiation to preserve epidermal homeostasis.

## Introduction

Circadian rhythm is an evolutionarily conserved time-keeping mechanism, allowing stem cells to know how and when to react to different signals inside and outside of the body. The circadian clock can be divided into two parts: the central clock, residing in the suprachiasmatic nucleus (SCN) of the hypothalamus, which receives light cues, and the peripheral clocks reside in various tissues throughout the body. These peripheral clocks play an integral and unique role within their respective tissues, driving the circadian expression of specific genes involved in a variety of physiological functions^1,2^. Circadian rhythms emerge progressively during fetal development and are initially immature and highly influenced by maternal signals. In humans, expression of core clock genes such as *BMAL1* and *PER2* has been detected in fetal tissues as early as the second trimester, although robust and self-sustained oscillations are typically not observed until late gestation or after birth^3,4^. The circadian clock responds to extrinsic environmental cues as well as intrinsic physiological and cell fate-determining processes. Different circadian gene signatures of biological processes exist in different tissues and adult stem cells^5,6^. These biological clocks maintain the homeostasis of adult stem cells. Disruptions of the circadian clock have been linked with various disease processes and aging^7–9^.

Skin serves as the body’s first defense, and skin diseases rank as the seventh leading global cause of years lived with disability^10,11^. The human epidermis is the outermost layer of the skin. Epidermal homeostasis is intricately maintained by the self-renewal and differentiation of epidermal stem and progenitor cells (EPSCs) located in the basal layer of the epidermis. These cells migrate upwards to become the suprabasal, granular, spinous, and cornified layers as they differentiate. Epidermal homeostasis relies on the precise temporal and spatial control of self-renewal and differentiation-associated gene expressions. Past research using primary neonatal keratinocytes has identified many transcriptional regulators and epigenetic regulators that are important for promoting human EPSCs self-renewal^12–15^ and inducing differentiation^16–20^. Due to an increase in the aging population and an unmet need of cell sources for allogeneic transplantation and generating grafts for clinical applications, fetal EPSCs could be an alternative since it has been previously characterized to have faster expansion time, longer telomeres, lower immunogenicity, and greater clonogenicity than adult keratinocytes^21^. However, the regulators of stemness in fetal EPSCs remain largely unexplored.

The structure and function of fetal epidermis differ substantially from the adult epidermis, highlighting the need to explore fetal skin characteristics as it relates to stem cell biology. During fetal skin development, at 4 weeks gestation, the skin is composed of two layers: the periderm and basal epidermis, with keratins K5 and K14 being expressed from 8 weeks of gestation. Other keratins, such as K9 and K19, are expressed during fetal skin development but are absent in the adult epidermis. At 9 and 10 weeks of gestation, keratinization becomes apparent and K1 and K10 are induced. At 14 weeks gestation, stratification of the epidermal layer is apparent, along with budding of the basal layer as the primordial hair follicle develops. Proteins involved in the formation of the cornified cell envelope, including involucrin, loricrin, and filaggrin, are detected from the time the stratum intermedium is formed^22,23^.

In the context of circadian regulation, previous rodent studies have shown that misregulation of circadian genes in stem cells results in premature epidermal aging and perturbed predisposition in skin tumorigenesis^24^. By comparing the young and aged mice epidermal stem cells transcriptome, dramatic changes in rhythmic gene profiles were observed, suggesting the significant involvement of circadian rhythm in the biological process of mouse epidermal aging^25^. However, the role of circadian genes in prenatal human epidermis remains largely unexplored, and further investigation into circadian regulation in fetal human epidermis could offer new insights into the developmental processes that regulate stem cell function and epidermal aging.

In this study, we profiled the rhythmic transcriptome of synchronized fetal and adult EPSCs and analyzed rhythmic gene expression using the Jonckheere–Terpstra–Kendall (JTK) algorithm. We identified common and unique patterns of cyclic genes and enriched pathways. Overall, the oscillating transcriptome showed a dampened amplitude in adult EPSCs compared to fetal ones. Common rhythmic genes in both fetal and adult EPSCs were enriched in cell cycle and DNA replication-related pathways. Genes with differing phase, amplitude, or mesor were enriched in Kyoto Encyclopedia of Genes and Genomes (KEGG) pathways related to cellular senescence, apoptosis, and TP53-mediated transcriptional regulation. Clustering unique rhythmic genes in fetal and adult EPSCs revealed distinct peak times for pro-proliferative and differentiation-associated genes during the 24-hour cycle. Promoter motif analysis of fetal rhythmic genes suggested enrichment of ZFP42, a YY1 family protein, as a potential upstream regulator. Interestingly, ZFP42 was highly expressed in fetal EPSCs, and its loss led to decreased proliferation and increased differentiation. Further data showed that ZFP42 maintains EPSC stemness by regulating the core clock gene *CRY1* expression. Knockdown of CRY1 resulted in a similar loss of proliferation and increase in differentiation in EPSCs as observed with ZFP42 knockdown. Overall, our study elucidates rhythmic gene expressions in human fetal and adult EPSCs and identifies ZFP42 as a stemness regulator, acting by regulating the core circadian gene *CRY1*. This work provides mechanistic insight into the coordinated regulatory network influencing adult stem cell stemness and circadian rhythm.

## Results

### Characterization of circadian periodicity in fetal and adult epidermal stem and progenitor cells

To verify whether the keratinocytes we isolated from fetal and adult skin tissues were epidermal stem and progenitor cells (EPSCs), we analyzed the expression of several well-known surface markers for human EPSCs, including CD49f^26^, CD34^27^, CD71^28^, and keratin 14 (a basal cell marker), using flow cytometry (Supplementary Fig. 1A, see Supplementary Data 4 for gating strategy). Our results showed that both groups of cells exhibited high expression levels of these markers. We also confirmed minimal expression of keratin 10 (an early differentiation marker) in both young and adult EPSCs (Supplementary Fig. 1B). Additionally, immunofluorescence staining for Mki67, along with the clonogenic assay, demonstrated that EPSCs from both fetal and adult skin tissues stained positively for Mki67 and retained the ability to form colonies (Supplementary Fig. 1C, D). These findings confirm that the isolated cells exhibit key characteristics of human EPSCs. Furthermore, our results align with previous studies that fetal EPSCs display enhanced stem cell-like properties, including higher expression of CD71 and Mki67, as well as larger colony sizes^21^.

**Fig. 1:**
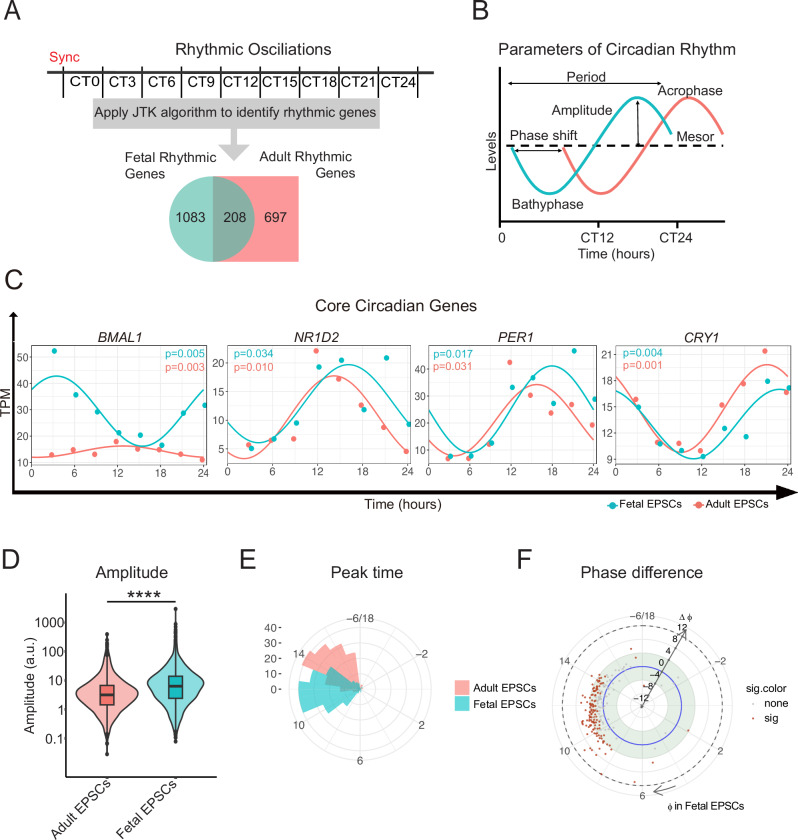
Distinct rhythmic transcriptome exists in fetal and adult EPSCs. **A** Scheme of the experimental design for profiling the rhythmic transcriptome. Passage 3 fetal and adult EPSCs (each sample was pooled from two biological replicates) were synchronized with a short serum pulse and harvested every 3 h within 24 h and transcriptome was profiled respectively. CT0 is the beginning of a subjective day. JTK algorithm was applied to detect rhythmic genes in fetal (teal) and adult (red) EPSCs, respectively and the Venn diagram shows the overlap of common rhythmic genes in both EPSCs. **B** Other parameters of circadian rhythm, including Amplitude, Mesor, and Phase shift, were also evaluated in both EPSCs using DiffCircaPipeline algorithm. **C** Representative core circadian genes, including *BMAL1*, *NR1D2*, *PER1*, and *CRY1*, exhibited robust rhythm in both fetal (teal) and adult (red) EPSCs. *p*-value was calculated using DiffCircaPipeline algorithm. Genes were considered cycling if *p* <0.05. The Y-axis represents normalized expression for each individual gene. **D** The amplitude of 208 common rhythmic genes was higher in fetal (teal) EPSCs than that in adult (red) EPSCs. **E** The 208 common rhythmic genes peaked at different times in fetal (teal) and adult (red) EPSCs. **F** Phase differences of 208 common rhythmic genes were shown between fetal and adult EPSCs. EPSCs, epidermal stem and progenitor cells; CT, circadian time. JTK Jonckheere–Terpstra–Kendall, TPM transcripts per million. ****p <0.0001 (Student’s *t* test).

To characterize the rhythmic genes in fetal and adult EPSCs, we initiated the process by synchronizing the cells with a brief serum pulse and subsequently harvested RNAs at intervals of 3 h within a 24-h period, as outlined in a previous study by Janich et al. ^29^. Following this, RNA-Seq was conducted to profile the transcriptome of these samples (Fig. 1A). Our results indicated that all samples of fetal and adult EPSCs expressed genes at similar levels and exhibited greater similarity to themselves by Principal Component Analysis (PCA) analysis (Supplementary Fig. 1E, F).

Subsequently, we employed the Jonckheere–Terpstra–Kendall (JTK) algorithm to identify rhythmic genes in both fetal and adult EPSCs, resulting in the detection of 1291 rhythmic genes in fetal EPSCs and 905 rhythmic genes in adult EPSCs, with an overlap of 208 genes^30^ (Supplementary Data 1A, B). These rhythmic genes also show significant overlap with findings from other studies in primary keratinocytes and human epidermis^29,31,32^ (Supplementary Data 2A, B). We also assessed circadian rhythm parameters including Phase, Amplitude, and Mesor using a comprehensive and interactive tool, DiffCircaPipeline (Fig. 1B and Supplementary Data 1A, B)^29,31–33^. Notably, several core clock genes, including *BMAL1*, *PER1*, *NR1D2*, and *CRY1*, showed significant rhythmicity in both sample sets, whereas *NR1D1* in adult EPSCs (*p* = 0.062) and *PER2* in fetal EPSCs (*p* = 0.065) exhibited only near-significant oscillatory patterns (Fig. 1C and Supplementary Fig. 1G). These findings support overall synchronization of the cultures, with gene-specific and context-dependent differences in the robustness of rhythmic oscillations. In both fetal and adult EPSCs, *BMAL1* and *CLOCK* displayed similar peak times at different phases, with a phase delay of ~12 h. Conversely, the peak times of *PER1* and *PER2* were antiphasic to *BMAL1*, in accordance with *BMAL1*’s known antiphasic relationship with its core clock targets^29^. Furthermore, a sequential pattern of peak times emerged, with *BMAL1* initially peaking around CT5 post-synchronization, followed by *NR1D1/2* and *CLOCK* peaking around CT10-CT15. Subsequently, *PER1/2* reached their peaks around CT15-CT20, and *CRY1/2* at CT20-CT24. Notably, *BMAL1* and *PER1/2* shared a similar period time of 24 h, while *CLOCK* and *NR1D1/2* exhibited longer period times exceeding 24 h. These findings indicate that the successive oscillation expression patterns of core clock components were generally conserved throughout human epidermal development.

Interestingly, we observed a notable reduction in the overall amplitude of rhythmic genes, in adult EPSCs when compared to fetal EPSCs. This suggests a dampening effect on circadian rhythm due to aging (Fig. 1D). In addition to this, the overlapped 208 rhythmic genes in adult EPSCs exhibited an average delay of ~3 h in peak times, and significant phase disparities were detected within the CT8–CT14 time frame (Fig. 1E, F). These findings underscore the existence of substantial disparities in rhythmic gene expression patterns between fetal and adult EPSCs, warranting further investigation.

### Exclusively rhythmic genes enriched critical biological processes in fetal and adult EPSCs

To comprehend the function of the conserved rhythmic genes between fetal and adult EPSCs, we generated a heatmap for these 208 overlapped genes, organized based on the order of fetal EPSCs (Supplementary Fig. 2A). Subsequently, we conducted Gene Ontology (GO) and KEGG analyses on the set of 208 overlapping genes. Both the GO terms and KEGG pathways revealed significant enrichment in terms such as DNA repair, DNA replication, cell cycle, and DNA synthesis, which are biological processes closely related to circadian regulation (Supplementary Fig. 2B).

To delve deeper into the differences between fetal and adult EPSCs regarding the Phase shift, Amplitude, and Mesor of these 208 rhythmic genes, we employed a comprehensive and interactive tool, DiffCircaPipeline^33^. Intriguingly, we identified 128 genes with significant phase shift differences between fetal and adult EPSCs, and these genes were enriched in GO terms and KEGG pathways related to antiviral mechanism by IFN-stimulated genes, defense response to virus, DNA damage response, and cell cycle (Supplementary Fig. 2C). The amplitude of these 208 genes exhibited significant overall differences (Supplementary Fig. 2D). Among them, 112 genes with altered amplitudes were enriched in GO terms associated with the cell cycle, DNA damage, and regulation of cellular senescence, as well as KEGG pathways such as the cell cycle, transcriptional regulation by *TP53*, and apoptosis (Supplementary Fig. 2E). Furthermore, 155 genes displayed differences in Mesor and were also in terms related to the cell cycle, DNA replication, and associated GO terms and KEGG pathways. Notably, GO terms like “S-adenosylmethionine-dependent methyltransferase”, “tricarboxylic acid (TCA) cycle”, “deubiquitination”, and “TP53 regulation of transcription of genes involved in G2 cell cycle arrest” were also observed (Supplementary Fig. 2F). Several rhythmic genes (*ARG2*, *BARD1, CLSPN*, *NEIL3*) common in fetal and adult EPSCs associated with cell senescence, apoptosis, DNA replication, and DNA repair are profiled here (Supplementary Fig. 2G). These findings suggest that essential biological processes are under the regulation of rhythmic genes during development, which may be conserved during adult stem cell aging.

**Fig. 2:**
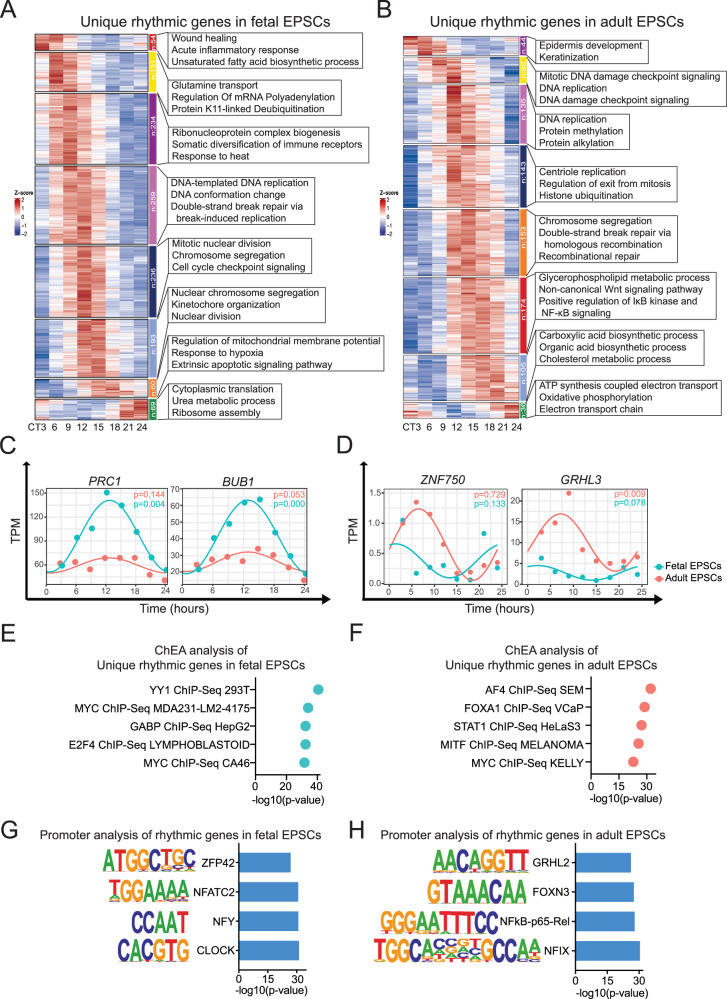
Unique rhythmic genes in fetal and adult EPSCs enriched critical biological processes. 1083 unique rhythmic genes in fetal (**A**) and 697 unique rhythmic genes in adult EPSCs (**B**) were clustered into 8 groups, respectively; their expressions were shown over time in the heatmap using ClusterGVi. GO of each group of genes was shown on the right side of the heatmap. **C** Representative rhythmic genes *PRC1* and *BUB1* are exclusively in fetal EPSCs (teal) and also shown in adult EPSCs (red). *p*-value was calculated using DiffCircaPipeline algorithm. **D** Representative rhythmic genes *ZNF750* and *GRHL3* are exclusively in adult EPSCs (red) and also shown in fetal EPSCs (teal). *p*-value was calculated using DiffCircaPipeline algorithm. **E** Top five enriched transcription factors were listed by ChEA analysis of unique rhythmic genes in fetal EPSCs. **F** Top five enriched factors were listed by ChEA analysis of unique rhythmic genes in adult EPSCs. **G** Promoter motif analysis of the rhythmic genes in fetal EPSCs by HOMER. Top motifs were listed by *p*-value. **H** Promoter motif analysis of the rhythmic genes in adult EPSCs by HOMER. Top motifs were listed by *p*-value. GO Gene Ontology, HOMER Hypergeometric Optimization of Motif EnRichment, ChEA ChIP Enrichment Analysis.

To elucidate the functional characteristics of the distinctive rhythmic genes with successive peaks in fetal and adult EPSCs, we employed hierarchical clustering for the time series genes and analyzed the enriched GO terms within each group using the R package ClusterGVi, respectively. As depicted in Fig. 2A, the earliest peak genes in fetal EPSCs were associated with GO terms related to wound healing and acute inflammatory responses. These were followed by genes linked to ribonucleoprotein complex biogenesis, the regulation of metabolic processes, and mRNA processing. Subsequently, a substantial cluster of genes (*n* = 478) related to DNA replication, mitotic nuclear division, and nuclear chromosome segregation exhibited peaks around CT6 to CT15. Afterward, genes associated with the regulation of mitochondrial membrane potential and cytoplasmic translation displayed their peaks.

In contrast, the initial group of genes with the earliest peaks in adult EPSCs was connected to epidermis development and keratinization at around CT3 (Fig. 2B). This was followed by genes related to DNA replication, nuclear division, and double-strand break repair (*n* = 338) that peaked from CT9 to CT15. Then, a cluster of 161 genes associated with protein localization to the nucleus and cell projection assembly showed peak expression at a similar time as another group of 175 genes linked to the non-canonical Wnt signaling pathway. Finally, GO terms encompassing carboxylic acid biosynthetic processes and unsaturated fatty acid biosynthetic processes, along with genes related to mitochondrial ATP synthesis coupled with electron transport, exhibited peaks around CT18 and CT24, respectively. Notably, the pattern where keratinocyte differentiation-related genes peaked earlier than genes associated with DNA replication and cell division (around late-night to early-morning hours vs. afternoon and evening) was consistent with earlier findings by Janich et al.^29^. The profiles of several representative rhythmic genes unique in fetal EPSCs and adult EPSCs were displayed. Cell division-related gene *PRC1* and mitosis-related gene *BUB1* are rhythmic only in fetal EPSCs. While epidermal differentiation-associated markers, including *ZNF750* and *GRHL3*, are rhythmic only in adult EPSCs (Fig. 2C, D). In contrast to the emphasis on biological functions in the DNA replication and cell cycle in fetal EPSCs, the functional segregation of pro-proliferative and differentiation cues enables these adult epidermal stem cells to maintain daily homeostasis and prepare for daylight UV radiation or external stressors.

To investigate the underlying molecular mechanisms that contribute to the differences in circadian rhythmicity between fetal and adult EPSCs, we performed the ChEA analysis for the unique rhythmic genes in each group. The analysis showed that transcription factors such as *YY1* and *GABP* are potential regulators of rhythmic gene expression in fetal EPSCs, while *AF4*, *FOXA1*, and *MITF* may exert a more prominent role in regulating rhythmic gene expression in adult EPSCs (Fig. 2E, F). It’s worth noting that MYC is an oncoprotein known to influence the expression of molecular clock genes, potentially disrupting the molecular clock oscillation in various cancer types^34^. Additionally, GABP proteins, characterized as 12-hour pacemakers in hepatic cells are significant transcription factors in this context^35^. Moreover, FOXA1 has recently been associated with its role in prostate cancer therapy as a downstream factor of *ARNTL*^36^. *MITF* has also been observed to exhibit rhythmic expression with a 24-hour periodicity in the presence of BMAL1^37^. The promoter motif analysis of the rhythmic genes in fetal EPSCs also confirmed the binding motif of YY1 family protein (ZFP42) as one of the top motifs enriched (Fig. 2G). In contrast, GRHL2 and NF-κB were among the top promoter motifs enriched in rhythmic genes of adult EPSCs (Fig. 2H). These findings indicate the existence of an intricate regulatory network involving transcription factors and their potential impact on circadian rhythmicity in EPSCs at different developmental stages in the skin.

### ZFP42 maintains human EPSCs self-renewal and inhibits differentiation

Next, we examined the significantly differentially expressed genes (DEGs) between fetal and adult EPSCs by comparing the paired time-series data from these two groups. Our analysis revealed a total of 691 genes exhibited high expression in fetal EPSCs, while 929 genes were highly expressed in adult EPSCs with a cutoff at a fold change ≥2 and a *p*-value < 0.05. The most enriched GO terms in fetal EPSCs included extracellular matrix organization, cell junction assembly, and collagen metabolic processes. In contrast, highly expressed genes in adult EPSCs demonstrated enrichment in GO terms like epidermal cell differentiation, epidermis development, and negative regulation of cell population proliferation (Supplementary Fig. 3A, B). To validate these findings, we performed qPCR on representative genes associated with extracellular matrix organization and epidermis development in multiple fetal, young and older adult EPSC donors (Supplementary Fig. 3C, D). These results suggest that young and older adult EPSCs are more differentiated with less proliferative potential than fetal EPSCs. Furthermore, upon examining the overlap between highly expressed genes and their respective rhythmic genes, we observed that 64 rhythmic and highly expressed genes in fetal EPSCs were enriched for cellular processes, developmental processes, and the regulation of biological processes (Supplementary Fig. 3E, F). In contrast, the 38 rhythmic and highly expressed genes in adult EPSCs were enriched for skin development, establishment of skin barrier, and immune response (Supplementary Fig. 3G, H). However, it’s essential to highlight that a substantial portion of the rhythmically expressed genes are not necessarily highly expressed. These findings further underscore the critical role of genes with circadian rhythm may be essential in the control of human epidermis development and epidermal function.

Among the top 100 DEGs between fetal and adult EPSCs, we observed the presence of ZFP42, a YY1 subfamily protein and well-known pluripotent transcription factor^38^ that was highly enriched in fetal EPSCs. In contrast, cell senescence gene *CDKN2A* and epidermal differentiation genes (*SPRR1B* and *IVL*) were highly expressed in adult EPSCs (Supplementary Fig. 3I). We further validated the ZFP42 expression in fetal, adult and old EPSCs from different donors using western blot and flow cytometry (Supplementary Fig. 4A–C, see Supplementary Data 4 for gating strategy). ZFP42, also known as Rex1, has been extensively studied as a pluripotent stem cell marker that is regulated by Oct3/4. More recently, it has been studied as a member of the YY1 subfamily. It was discovered to originate from a retroposition duplication of YY1 in eutherian mammals, and they share some similarities at their core regions^39^. Our ChEA analysis and promoter motif analysis of the fetal rhythmic genes showed significant enrichment for ZFP42 binding sites, which suggests that it may regulate the unique rhythmic genes in fetal EPSCs (Fig. 2E, G). Thus, we suspect that ZFP42 could potentially be involved in the circadian regulation of fetal EPSCs.

**Fig. 3:**
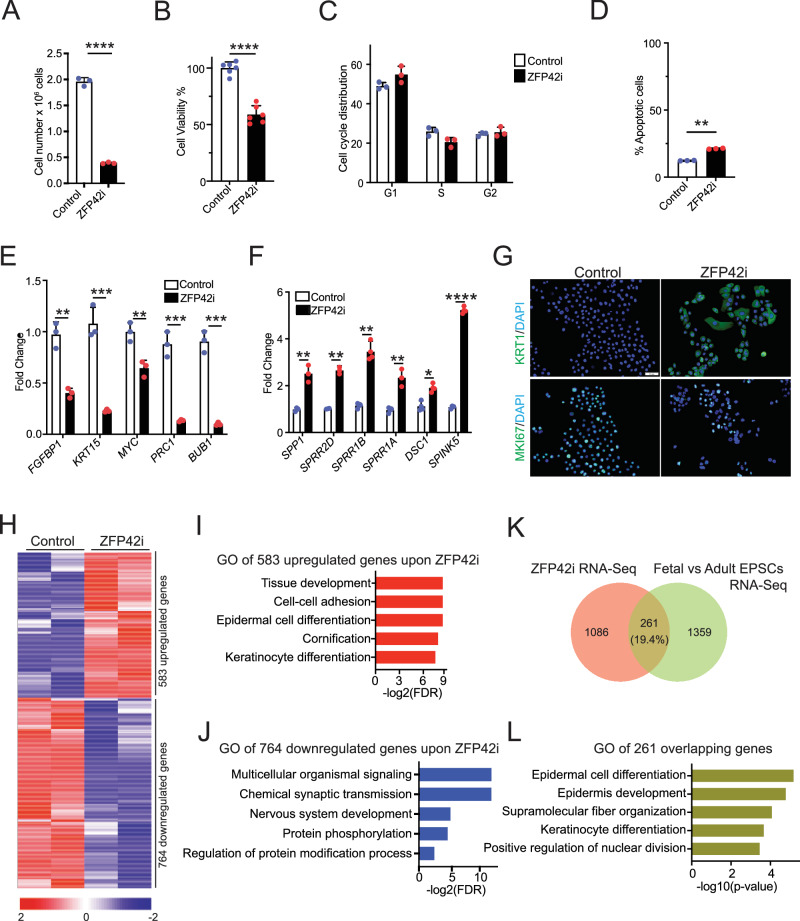
ZFP42 is a stemness regulator in human EPSCs. **A** Proliferating EPSCs derived from young children were knocked down with control (*n* = 3) and ZFP42 siRNAs (*n* = 3) and plated at the same density at day 0. Cell number was counted 3 days after transfection. **B** Cell viability of Control (*n* = 6) and ZFP42 knockdown (ZFP42i) young EPSCs (*n* = 6) measured by CCK8 assay at 3 days post-transfection. **C** Cell cycle analysis of Control (*n* = 3) and ZFP42i (*n* = 3) young EPSCs at 3 days post-transfection. **D** Apoptosis analysis of Control (*n* = 3) and ZFP42i young EPSCs (*n* = 3) at 3 days post-transfection. **E** RT-QPCR for detecting epidermal proliferation gene (*FGFBP1, KRT15, MYC, PRC1* and *BUB1*) expressions in Control (*n* = 3) and ZFP42i (*n* = 3) young EPSCs. QPCR results were normalized to L32 levels. **F** RT-QPCR of differentiation genes (*SPP1, SPRR2D, SPRR1B, SPRR1A, DSC1* and *SPINK5*) in the Control (*n* = 3) and ZFP42i (*n* = 3) young EPSCs at 3 days post-transfection. **G** Expression of proliferation marker MKi67 and differentiation marker Keratin 1 (KRT1) in Control (*n* = 3) and ZFP42i young EPSCs (by siRNA) (*n* = 3) was characterized by immunostaining at day 3. Representative image is shown. **H** RNA-Seq analysis of Control (*n* = 2) and ZFP42i fetal EPSCs (*n* = 2) using *ZFP42* shRNA harvested 3 days after infection. Heatmap of genes that change upon ZFP42 depletion is shown in red (upregulated upon ZFP42i) and blue (downregulated upon ZFP42i) on a log2 scale. **I** GO terms of the 583 upregulated genes in ZFP42i cells using Enrichr. **J** GO terms of the 764 downregulated genes in ZFP42i cells using Enrichr. **K** Venn diagram of overlapped genes between ZFP42i RNA-Seq and DEGs between fetal and adult EPSCs. **L** GO analysis of the overlapped 261 genes using Enrichr. *p <0.05, **p <0.01, ****p <0.0001 (Student’s *t* test). *N* is a minimum of at least 3. Mean values are shown with error bars representing standard deviations (SD). GO Gene Ontology, RT-QPCR reverse transcription quantitative PCR, siRNA small interfering RNA, shRNA short hairpin RNA, DEG differentially expressed genes.

Due to limited availability of fetal EPSCs, we proceeded to perform ZFP42 knockdown experiments in young EPSCs (derived from young children) using siRNAs or shRNAs. Depletion of ZFP42 significantly inhibited cell growth and decreased cell viability as indicated by cell proliferation and cell viability measured by CCK8 assay (Fig. 3A, B and Supplementary Fig. 4D). Cell cycle analysis also showed that the knockdown of ZFP42 resulted in an accumulation of cells in the G1 phase and a decrease of cells in the S phase, suggesting DNA synthesis in the ZFP42 knockdown cells was significantly reduced (Fig. 3C, see Supplementary Data 4 for gating strategy). Knockdown of ZFP42 also increased cell apoptosis (Fig. 3D, see Supplementary Data 4 for gating strategy). Furthermore, a decrease in the expression of proliferation-related genes, such as *FGFBP1*, *KRT15*, *MYC*, *PRC1*, and *BUB1*, was observed (Fig. 3E). Meanwhile, the knockdown of ZFP42 promoted the expression of differentiation-related genes, including *SPRR1A*, *SPRR1B*, and *SPINK5* (Fig. 3F). Immunostaining also confirmed the increase of differentiation protein, Keratin 1, and the decrease of MKI67 positive cells in ZFP42 knockdown cells (Fig. 3G and Supplementary Fig. 4E).

To gain a deeper understanding of the molecular changes resulting from ZFP42 knockdown in fetal EPSCs, we conducted RNA-Seq analysis to profile the transcriptome alterations. As expected, we found that 583 upregulated genes were enriched for processes related to tissue development, epidermal cell differentiation, and cornification, while 764 downregulated genes were associated with multicellular organism signaling and chemical synaptic transmission (Fig. 3H–J and Supplementary Data 3A). Remarkably, approximately 20% of these DEGs following ZFP42 knockdown overlapped with DEGs observed when comparing fetal and adult EPSCs (Fig. 3K). This set of 261 overlapping genes was enriched in functions related to epidermal cell differentiation, epidermis development, and positive regulation of nuclear division (Fig. 3L).

**Fig. 4:**
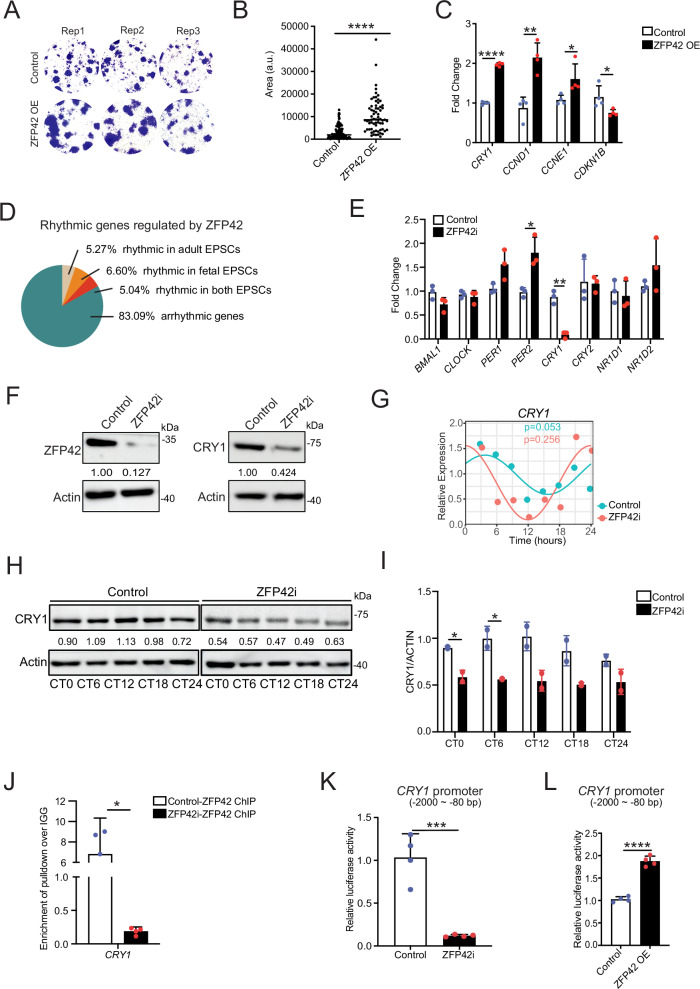
ZFP42 regulates EPSCs stemness through sustaining CRY1 expression and its rhythmicity. **A** Colony formation assay of Control (*n* = 3) and ZFP42 overexpressed (*n* = 3) EPSCs derived from older adults. ZFP42 OE is overexpression of ZFP42. **B** Quantification of colony formation assay. **C** Measurement of cell cycle-associated genes upon ZFP42 OE in EPSCs derived from older adults. **D** Overlap of ZFP42-regulated genes (RNA-Seq) with rhythmic genes (data in Fig. 1A) in fetal and adult EPSCs. **E** Measurement of core circadian rhythm genes (*BMAL1, CLOCK, PER1/2, CRY1/2*, and *NR1D1/2*) in Control (*n* = 3) and ZFP42 knockdown young EPSCs (ZFP42i) (*n* = 3) using siRNAs. **F** Measurement of ZFP42 and CRY1 at protein levels in Control (*n* = 3) and ZFP42i young EPSCs (*n* = 3). Representative image is shown. **G** CRY1 expression over time upon knockdown of ZFP42 in Control (*n* = 3) and ZFP42i young EPSCs (*n* = 3) at mRNA levels. **H** Measurement of CRY1 expression over time upon knockdown of ZFP42 in young EPSCs. Representative image is shown, *N* = 3 independent experiments. **I** Quantification of western blot results by calculating the ratio of CRY1 to beta-actin. **J** ChIP-qPCR of ZFP42 pulldown in both Control (*n* = 4) and ZFP42i young EPSCs (*n* = 4). IGG pulldowns in Control and ZFP42i cells were used as a negative control. qPCR was used to determine the amount of binding to genes listed on the X-axis. Results are plotted as fold change over IGG. *CRY1* primer was targeted toward the TSS (-420 to -172 bp) of *CRY1* gene. *N* = 3 independent experiments. **K** Measurement of *CRY1* promoter activity in both Control (*n* = 4) and ZFP42i fetal EPSCs (*n* = 4) using a dual-luciferase reporter assay. **L** Measurement of *CRY1* promoter activity in both Control (*n* = 4) and ZFP42 overexpressed fetal EPSCs (*n* = 4) using a dual-luciferase reporter assay. ZFP42 OE is overexpression of ZFP42. *p <0.05, **p <0.01, ***p <0.001, ****p <0.0001 (Student’s *t* test). N is a minimum of at least 3.Mean values are shown with error bars representing standard deviations (SD). OE overexpression.

More importantly, overexpression of ZFP42 rejuvenated the colony formation abilities in EPSCs from older adults (harvested from subjects ≥ 60 years old) (Fig. 4A, B), demonstrating ZFP42’s pivotal role as a stemness regulator in human EPSCs. Restoration of ZFP42 exprtession also rescued the expression of cell cycle-associated genes (Fig. 4C). Notably, however, *ZFP42* itself is not rhythmic in either fetal (*p* = 0.080) or adult EPSCs (*p* = 0.509) (Supplementary Fig. 4F). In fetal EPSCs, *ZFP42* showed modest positive correlations *KLF4* (*r* = 0.31, *p* = 0.404) and *NANOG* (*r* = 0.31, *p* = 0.415), whereas no meaningful correlation was detected in adult EPSCs (*KLF4*: *r* = −0.06, *p* = 0.869; *NANOG*: *r* = −0.02, *p* = 0.961) (Supplementary Fig. 4G). Because these *p*-values exceed the conventional significance threshold, we interpret these trends cautiously and do not claim statistical significance. Together, our result suggests that ZFP42 may influence fetal EPSCs’ stemness genes by regulating clock genes rather than possessing inherent rhythmicity.

### CRY1 is a downstream target of ZFP42 that promotes EPSCs proliferation and inhibits differentiation

We then closely examined the rhythmic genes that were regulated by *ZFP42*, and interestingly, we found that 5.27% of genes were rhythmic in adult EPSCs, while 6.60% were rhythmic in fetal EPSCs. Additionally, 5.04% of genes were rhythmic in both sample sets, including the core circadian gene *CRY1* (Fig. 4D). Furthermore, we assessed the expression of core clock genes (*BMAL1*, *CLOCK*, *PER1/2*, *CRY1/2*, and *NR1D1/2*) following ZFP42 knockdown and discovered that only CRY1 exhibited significant downregulation at both the mRNA and protein levels (Fig. 4E, F). Also, the decrease of *CRY1* upon *ZFP42* knockdown was not time-specific (Supplementary Fig. 4I). Consistent with our hypothesis that ZFP42 transcriptionally regulates *CRY1*, we observed a significant positive linear correlation between *ZFP42* and *CRY1* expression in fetal EPSCs (*r* = 0.49, *p* = 0.168), whereas no correlation was detected in adult EPSCs (*r* = −0.14, *p* = 0.717) (Supplementary Fig. 4G). Although the fetal correlations do not meet the *p* < 0.05 criterion, we report it as a positive biological trend that requires further validation with an increased sample size. For completeness, Spearman’s rank correlation is also provided in the panel (Supplementary Fig. 4H). Knockdown of ZFP42 also disrupts the rhythmicity of *CRY1* and *CLSPN* in EPSCs, which is similar to changes (increase in amplitude and a phase shift) from fetal to adult EPSCs (Fig. 4G–I, and Supplementary Fig. 4J). We also confirmed the binding of ZFP42 to the promoter region (–420 to –172 bp upstream of transcription start site) of *CRY1* by ChIP-QPCR (Fig. 4J). A promoter activity assay using a Dual-Luciferase reporter system was conducted to analyze *CRY1* promoter activity in both ZFP42 knockdown and overexpression cells, as well as their respective controls (Fig. 4K, L). The results showed a significant reduction in *CRY1* promoter activity following ZFP42 knockdown, whereas its activity increased with ZFP42 overexpression. As a positive control, we used *CCNB1*, a previously reported ZFP42 target gene^40^, to validate the assay (Supplementary Fig. 4K, L). These findings suggest that CRY1 may act downstream of ZFP42, functioning as a potent stemness regulator in human EPSCs.

**Fig. 5:**
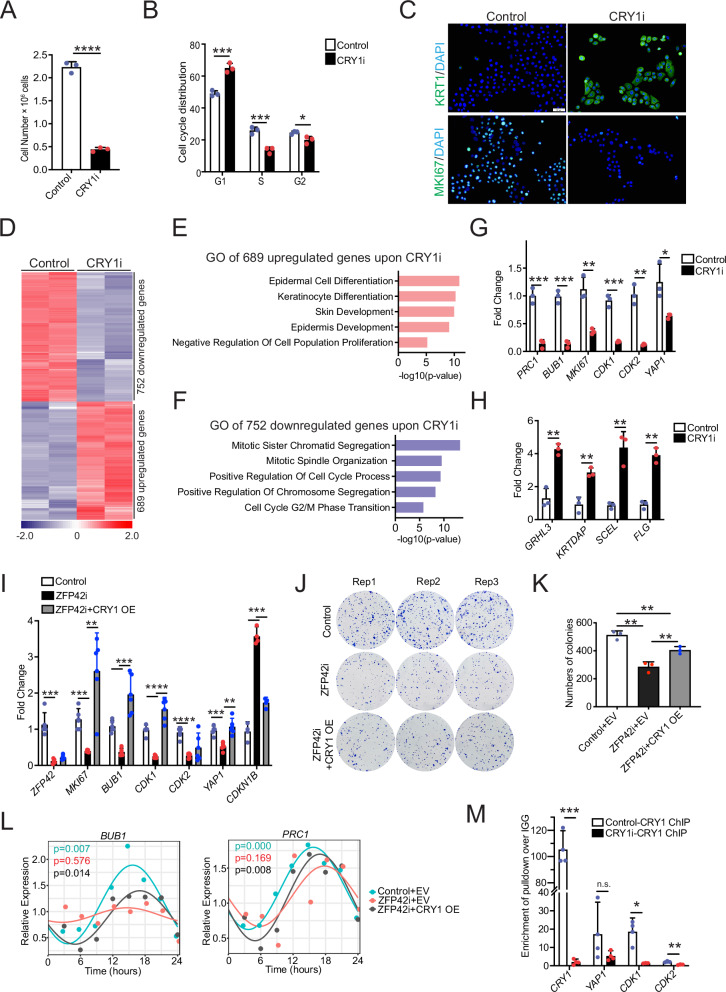
CRY1 is essential for maintaining the homeostasis of self-renewal and differentiation in human EPSCs. **A** Cell number was counted after transfection of Control (*n* = 3) and CRY1 siRNAs (*n* = 3) in young EPSCs after 3 days. **B** Cell cycle analysis of Control (*n* = 3) and CRY1 knockdown (CRY1i) young EPSCs (*n* = 3) at 3 days post-transfection. **C** Immunofluorescence staining of proliferation marker MKi67 and differentiation marker KRT1 in Control (*n* = 3) and CRY1i young EPSCs (*n* = 3) at 3 days post-transfection. Representative image is shown. **D** RNA-Seq analysis of Control (*n* = 2) and CRY1i young EPSCs (*n* = 2) harvested 3 days after transfection. Heatmap of genes that change upon CRY1 depletion is shown in red (upregulated upon CRY1i) and blue (downregulated upon CRY1i) on a log2 scale. **E** GO analysis of 689 upregulated genes upon CRY1 knockdown using Enrichr. **F** GO terms of the 752 downregulated genes upon CRY1 knockdown using Enrichr. **G**, **H** CRY1 knockdown resulted decrease in cell proliferation genes (*PRC1*, *BUB1*, *MKI67*, *CDK1*, *CDK2*, and *YAP1*) and an increase in differentiation genes (*GRHL3*, *KRTDAP*, *SCEL*, and *FLG*). *N* = 3 independent experiments. **I** RT-QPCR quantifying the mRNA expression of proliferation-associated genes (*MKI67, BUB1, CDK1, CDK2, YAP1* and *CDKN1B*) in samples including Control+EV (*n* = 5), ZFP42i + EV (*n* = 5), and ZFP42i + CRY1 OE (*n* = 5) young EPSCs. EV is empty vector and CRY1 OE is overexpression of CRY1. RT-QPCR values were normalized to L32. **J** Colony formation assay of Control + EV, ZFP42i + EV, and ZFP42i + CRY1 OE young EPSCs. **K** Quantification of number of colonies in colony formation assay. **L** Expressions of *BUB1* and *PRC1* over time in Control+EV, ZFP42i+EV, and ZFP42i + CRY1 OE young EPSCs. **M** CRY1 ChIP-qPCR on Control (*n* = 4) and CRY1i young EPSCs (*n* = 4). IGG pulldowns in control and CRY1i cells were used as a negative control. qPCR was used to determine the amount of binding to genes listed on the X-axis. Primers were targeted toward the TSS of each gene. Results are plotted as fold change over IGG. *N* = 3 independent experiments. *p <0.05, **p <0.01, ***p <0.001, ****p <0.0001 (Student’s *t* test was performed for comparison between two groups and one-way ANOVA followed by Tukey’s multiple comparison for 3 groups). Mean values are shown with error bars representing standard deviations (SD). GO Gene Ontology, DEG differentially expressed genes, RT-QPCR reverse transcription quantitative PCR, KRT1 Keratin 1, EV empty vector, OE overexpression.

To investigate this, we knocked down CRY1 using three independent siRNAs. Similar to the effects of ZFP42 knockdown, the loss of CRY1 significantly inhibited cell growth and promoted epidermal differentiation (Fig. 5A and Supplementary Fig. 5A–C). Notably, cell cycle analysis indicated that the knockdown of CRY1 led to an accumulation of DNA synthesis in the G1 phase and a decrease in the S and G2 phases (Fig. 5B, see Supplementary Data 4 for gating strategy). Immunofluorescence staining of CRY1 knockdown cells confirmed an increased expression of epidermal differentiation marker Keratin 1 and a decreased expression of Mki67, mirroring the effects observed in ZFP42 knockdown cells (Fig. 5C and Supplementary Fig. 5D). Furthermore, our RNA-Seq analysis of CRY1 knockdown cells identified a total of 689 upregulated genes and 752 downregulated genes, with a cutoff at a fold change >=2 and a *p*-value < 0.05. The GO analysis of the upregulated genes indicated enrichment in terms related to epidermal cell differentiation, keratinocyte differentiation, and skin development. Conversely, the downregulated genes were enriched in GO terms like mitotic sister chromatid segregation, mitotic spindle organization, and positive regulation of the cell cycle process (Fig. 5D–F and Supplementary Data 3B). A significant portion (24.3%) of the DEGs resulting from CRY1 knockdown, also overlapped with DEGs observed when comparing fetal and adult EPSCs (Supplementary Fig. 5E). Once again, these 350 overlapped genes were enriched in processes related to epidermal development, extracellular matrix organization, and the regulation of cell population proliferation (Supplementary Fig. 5F). Collectively, these findings indicate the significant role of *CRY1*, a core circadian gene, in maintaining the self-renewal and differentiation of epidermal stem cells.

To validate, QPCR was employed to confirm the decreased expression of the cell cycle-related genes, such as *MKI67*, *PRC1*, and *BUB1*, and the increase of the differentiation-inducing genes and markers, including *GRHL3*, *KRTDAP*, *SCEL*, and *FLG* upon CRY1 knockdown (Fig. 5G, H). By comparing the DEGs between ZFP42i and CRY1i RNA-Seq, we found 171 (~13%) genes overlapped (Supplementary Fig. 5G). Moreover, overexpression of CRY1 did not alter the expression of ZFP42 but was able to rescue the decrease in the expression of proliferation gene expressions resulting from ZFP42 knockdown, including *MKI67*, *BUB1*, *CDK1, CDK2*, and *YAP1* (Fig. 5I and Supplementary Fig. 5H). To confirm the rescue of ZFP42 knockdown phenotype by CRY1 overexpression, we performed colony formation, cell cycle analysis, and immunostaining for Mki67. The results showed that CRY1 overexpression partially restored the EPSCs stemness, as evidenced by the increased number of colonies, a higher percentage of Mki67 positive cells and an increase of S phase, consistent with a decrease of *CDKN1B* expression (Fig. 5I–K, Supplementary Fig. 5I–K, see Supplementary Data 4 for gating strategy). However, it did not prevent the increase in differentiation genes upon ZFP42 knockdown (Supplementary Fig. 5L). These findings suggest that CRY1 plays a more prominent role in regulating cell proliferation rather than differentiation in EPSCs.

Furthermore, we analyzed the rhythmic genes that were influenced by *CRY1* manipulation and found that 14.22% of the DEGs were rhythmic in fetal EPSCs, compared to 10.51% in adult EPSCs, with 13.8% displaying rhythmicity in both fetal and adult EPSCs (Supplementary Fig. 5M). In total, ~40% of the DEGs upon CRY1 knockdown exhibited rhythmic patterns, underscoring the crucial role that core clock genes play in the biological processes related to human epidermis homeostasis. Interestingly, restoring CRY1 expression in ZFP42 knockdown cells rescued the rhythmicity of proliferation genes (*BUB1* and *PRC1*), but did not restore rhythmicity for differentiation-inducing genes (*ZNF750* and *GRHL3*) (Fig. 5L, Supplementary Fig. 6A). Our data suggest that ZFP42 regulates circadian output partly through CRY1 and partly via CRY1-independent pathways.

Recent studies have characterized the chromatin binding of CRY1 in both human and mouse genomes. These studies also demonstrated non-traditional role of CRY1 in terms of regulating and reprogramming induced pluripotent stem cells and DNA repair in cancer^41–43^. We were able to identify CRY1 binding to the promoter regions of proliferation genes such as *CDK1*, *CDK2*, and *YAP1* in both human and mouse datasets (Supplementary Fig. 6B, C). We further validated these binding sites by ChIP-QPCR in both control and CRY1 knockdown cells (Fig. 5M). In summary, our data suggested that CRY1 is regulated by ZFP42 and also functions in maintaining proliferating genes and inhibiting differentiation in human EPSCs.

## Discussion

To identify stemness regulators potentially involved in circadian rhythm mechanisms in human EPSCs, we employed fetal and adult EPSCs as models and profiled their rhythmic transcriptome after synchronization. Our results indicated that the oscillation of the core clock transcriptional machinery and hundreds of other transcripts exists in both fetal and adult EPSCs. The successive waves of genes associated with critical biological processes go through different peaks during the 24-h day cycle. Rhythmic genes in fetal EPSCs are possibly regulated by ZFP42, which is expressed highly in fetal EPSCs, and functions as a stemness regulator through regulating CRY1 expression, supporting that stem cell functions are deeply implicated by circadian rhythm.

A previous study investigated the aging and circadian rhythm and found that the amplitude of rhythmic genes declined with aging in vivo. They postulated that the stem cell niche functional decline leads to the decreased ability to respond to SCN cues^25^. An in vivo study comparing average amplitude of oscillations of the daily rhythmic genes found no difference between adult and aged mouse epidermal and muscle stem cells, suggesting a possible difference between human and other mammals impacted by the living environment or the lifespan^25^. In our study, a higher amplitude of rhythmic genes occurs in the fetal EPSCs compared to adult EPSCs also suggesting aging decreases the amplitude of rhythmic genes. Core clock genes remain equally and robustly oscillated in both fetal and adult EPSCs. However, since our experiments were conducted in vitro without signals from SCN, it is also possible that the stemness programs controlled by transcription factors such as ZFP42 decline with aging, resulting in less responsive rhythmicity for downstream rhythmic genes, suggesting a potential mechanism of cell-intrinsic decline in amplitude of rhythmic genes regardless of SCN signals. Interestingly, studies in other fetal tissues have shown that fetal circadian clocks are immature but display higher plasticity and, in some cases, stronger oscillatory capacity under specific environmental cues compared to adult tissues^44–46^. Our observation that fetal EPSCs exhibit higher circadian amplitude under in vitro conditions suggests potential developmental differences in clock responsiveness; however, this may not reflect intrinsic clock activity in vivo. As robust circadian rhythms are generally thought to emerge later in development^3,4^, future studies using in vivo fetal tissue will be critical to determine whether a functional epidermal clock is active at this stage.

The involvement of WNT signaling in the circadian regulators (BMAL1 or REV-ERBα) regulates cell division or replication has been reported in several types of adult stem cells, including satellite cells, intestinal epithelial cells^47–49^. In this study, we also found that the GO terms of Non-canonical Wnt signaling pathway and Positive regulation of IκB kinase and NF-κB signaling are enriched in the rhythmic genes peaked around CT15 and CT18 in adult EPSCs. It has been reported that non-canonical Wnt signaling can inhibit canonical Wnt signaling in certain context^50^, which may affect hair follicle formation and wound healing. Additionally, NF-κB signaling is associated with inflammation. The increase of NF-κB activity provides protection against cell apoptosis in the homeostatic condition^51,52^. Consistently, promoter analysis of rhythmic genes in adult EPSCs also exhibited enrichment of NF-κB. This may explain the decline of the adult stem cell functions and shift of rhythmic genes associated with aging and inflammation.

Yin-Yang 1 (YY1) has two independently retroposed copies, YY2 and Reduced Expression 1 (REX1, also known as ZFP42). YY1 is recently characterized as a key regulator of multidimensional epigenetic crosstalk associated with extended pluripotency in embryonic stem cells (ESCs)^53^. ZFP42’s sequence is very similar to YY1 and it has been well studied as a stem cell marker in ESCs^38^. We analyzed the possible upstream regulators for the rhythmic genes in fetal EPSCs and found an enriched motif for ZFP42, which is also highly expressed in fetal EPSCs. Our results suggest that ZFP42 maintains human EPSCs self-renewal and prevents epidermal differentiation by maintaining the expression of circadian regulator CRY1.

Our findings suggest that ZFP42 knockdown alters CRY1 expression in a time-dependent manner. While CRY1 expression decreased, its oscillation amplitude increased, indicating that loss of ZFP42 disrupts the normal circadian feedback loops, leading to larger fluctuations in expression. This could reflect an initial reduction in CRY1 levels followed by compensatory mechanisms that increase amplitude. Since CRY1 represses CLOCK/BMAL1 activity, its reduction weakens this inhibition and enhances CLOCK/BMAL1-driven transcription of downstream targets, including *PER1* and *PER2*. Accordingly, *PER1* and *PER2* levels increase (*p* = 0.055 and 0.014, respectively) upon ZFP42 knockdown, consistent with decreased CRY1-mediated repression. These opposing changes in *CRY1* versus *PER1*/*PER2* observed upon ZFP42 knockdown align with the canonical circadian feedback loop. These dynamics were further supported by the changes observed in circadian expression patterns during rescue experiments. Future studies with longer time points and multiple cycles and rescue experiments calibrated to physiological levels will be essential to clarify ZFP42’s role in regulating circadian rhythm and its impact on oscillation amplitude and expression levels of key clock genes like *CRY1*.

Although ZFP42 is highly conserved among vertebrates, analyses of murine and multi-tissue human circadian datasets did not reveal rhythmic expression^25,54^, suggesting its contribution to CRY1 regulation in human epidermal progenitors may represent a tissue- or species-specific adaptation. Meanwhile, our findings suggest that the requirement for ZFP42 in regulating CRY1 expression is not a universal feature of circadian control but instead reflects a developmental-stage-specific role in fetal epidermis. Circadian rhythms are well established in many mature tissues that do not express ZFP42, indicating that alternative mechanisms sustain *CRY1* expression in differentiated contexts. We did not perform murine cross-species experiments to separate developmental context from circadian control; evolutionary conservation of the ZFP42-CRY1 axis therefore remains untested.

CRY1 loss resulted in a significant decrease in cell proliferation, highlighting the importance of circadian control in epidermal development before birth. The role of CRY1 has also been demonstrated in the context of pluripotent stem cells (PSCs), suggesting that CRY1 is required for the maintenance of self-renewal capacity, colony organization, and metabolic signatures, and cells lacking CRY1 exhibit differential gene expression profiles during induced PSCs reprogramming^42^. Another study showed that the reduction of CRY1 induced apoptosis of p53 knockout fibroblasts^55^. Our study provides some mechanistic understanding of the link between the molecular clock and adult stem cell stemness, which may help decipher the molecular insights into skin aging and related diseases.

CRY1 binds to genes related to the mitotic cell cycle in pluripotent stem cells and mouse embryonic fibroblasts. Interestingly, Cry1 knockout also resulted in an increase of ectoderm lineage factor Sox1 expression while decreasing most of the other lineage differentiation markers in ESCs. The non-canonical roles of CRY1 during induced pluripotent stem cells reprogramming are associated its metabolic reprogramming function by inactivating AMPK and activating SREBP1^42^. The versatile and diverse functions of CRY1 could be achieved through its interaction with many nuclear receptors and transcription factors at the transcriptional level and downstream signaling^56–58^.

Our CRY1 rescue experiment also showed that CRY1 re-expression in ZFP42-depleted cells partially restores circadian rhythms of proliferation genes and cell-cycle progression, but does not recover differentiation markers. This suggests that ZFP42’s regulation of CRY1 is specifically important for circadian control of cell division, but that ZFP42 may also engage additional pathways necessary for differentiation, which are independent of CRY1. These findings highlight a differential role of CRY1 in proliferative vs. differentiation gene regulation and emphasize the complexity of ZFP42’s function in maintaining progenitor state. Additionally, previous study showed that ZFP42 could activate p38 MAPK to regulate the proliferation through inducing cell cycle arrest in human mesenchymal stem cells^59^. Our preliminary data suggested that ZFP42 loss activated p38 MAPK pathway but not rescued by CRY1 overexpression, suggesting that ZFP42 may have a broader role in regulating cell cycle and cell proliferation, which is independent of CRY1-mediated circadian rhythm in EPSCs. However, potential CRY1-independent MAPK effects were not quantified, and the precise placement of the related pathways (e.g. stress/senescence) within the regulatory network remains to be defined systematically.

Our data suggest that ZFP42 contributes to circadian regulation in fetal EPSCs through both direct and permissive mechanisms. Although ZFP42 itself is non-rhythmic, ChIP-qPCR and reporter assays demonstrate that it binds to and activates the *CRY1* promoter, supporting a direct regulatory role. Consistent with this, knockdown of ZFP42 reduced CRY1 expression and, importantly, abolished its rhythmicity, indicating that ZFP42 is required for circadian oscillation of CRY1. These findings imply that ZFP42 does not act as a canonical oscillator but rather provides an essential regulatory input that enables CRY1 to cycle. We propose that ZFP42 functions by both directly engaging the CRY1 regulatory region and by maintaining a transcriptionally competent state in fetal EPSCs that permits circadian gene expression. While this study demonstrates ZFP42’s direct interaction with CRY1, further validation using genome-wide ChIP-Seq and promoter mutation assays is needed. However, due to the limited availability of fetal EPSCs, we were unable to perform these additional experiments. The exact ZFP42 binding site in the CRY1 promoter remains undefined, preventing targeted mutation assays. Whether ZFP42 acts permissively via chromatin/stemness, directly on clock genes, or both remains unresolved. Future studies, with expanded biological replicates and more precise genomic techniques, will be crucial to confirm ZFP42’s role as a transcriptional regulator of CRY1 and to further elucidate its impact on circadian gene regulation in epidermal stem cells.

Our analysis of publicly available ZFP42 ChIP-Seq data from mouse ESCs identified significant peaks near the CRY1 promoter, suggesting that ZFP42 might also play a conserved role in circadian regulation in pluripotent stem cells (Espejo-Serrano et al., 2024). Whereas ZFP42 is known to support pluripotency without regulating circadian rhythms, our data indicate it contributes to circadian competence in EPSCs, potentially through lineage-specific targets such as CRY1. Developmental repurposing of ZFP42 for circadian control is inferred but unproven without comparative binding maps comparisons across pluripotent, young, and aged epidermal cells, nor by promoter-element perturbation. Further experiments comparing ZFP42 ChIP-Seq analyses across pluripotent stem cells, young and aged EPSCs will be essential to define this mechanistic divergence and provide insights into ZFP42’s lineage-specific role in circadian regulation.

In conclusion, we profiled the circadian rhythmic transcriptome in fetal and adult EPSCs in vitro for the first time. Moreover, we further identified a pluripotent factor ZFP42, and characterized its regulatory role in human EPSC self-renewal and differentiation. We also demonstrated that ZFP42 regulates the core circadian clock gene *CRY1*. Collectively, our results establish a functional ZFP42 - CRY1 pathway driving proliferation-linked rhythmic outputs, even as developmental repurposing and evolutionary generality remain to be mapped.

Although our in vitro culture system enabled direct comparison of circadian differences between fetal and adult EPSCs, we recognize that culture may alter the native properties of epidermal stem cells. Our circadian analyses are limited to a single 24-h serum-shock window without multi-cycle, constant-condition recordings or FDR-controlled rhythmicity thresholds. Time-course RNA-seq used pooled donors and small n, which may impact gene-level rhythm calls and amplitudes. Therefore, donor-resolved temporal datasets will be required, and future in vivo studies will be essential to validate whether the observed circadian differences also exist in the physiological epidermal niche.

Our analyses were conducted using the hg19/GENCODE v19 reference genome, and re-analysis on the updated GRCh38 build could improve annotation accuracy and consistency. Additionally, the rhythmicity assessment relied on JTK_CYCLE results without FDR correction, and thus only *p*-values—not *q*-values—were reported. Finally, although time-series data were evaluated using standard t-tests and ANOVA, more robust approaches such as mixed-effects or cosinor models with confidence intervals were not implemented, which may limit the precision of temporal and rhythmic inferences. Another limitation is that the collection times were not consistently recorded, and the number of samples per time point was small. Incorporating accurate time-of-day metadata, particularly for abortion specimens, could offer valuable insight into in vivo circadian dynamics and will be included in future study designs.

This study is also limited by relatively small sample sizes and a pooling strategy, particularly in experiments involving fetal skin-derived EPSCs. Due to constraints such as the difficulty in obtaining samples during the COVID-19 pandemic, only a limited number of biological replicates were available. While we attempted to mitigate variability by pooling samples and performing comprehensive analyses, we acknowledge that the pooling strategy used for fetal EPSCs, while necessary due to sample scarcity, does not fully account for inter-donor variability. This limitation may reduce the statistical power and contribute to variability in the detection of circadian genes and amplitude differences between fetal and adult EPSCs. Because inter-donor rhythmicity and amplitude require independent, donor-resolved temporal datasets, our findings should be interpreted with caution. Future studies with larger donor cohorts and donor-level time-series profiling will be critical to independently validate circadian transcription in human epidermal stem cells. Larger sample sizes would strengthen the robustness of our conclusions and provide more reliable insights into the broader population variability. Future in vivo studies will be essential to validate our findings and extend the understanding of ZFP42 and CRY1’s roles in circadian regulation and stemness in human EPSCs.

## Methods

### Isolation of human epidermal stem and progenitor cells

Primary human epidermal keratinocytes were isolated from the discarded foreskin of young children (aged between 5–10 years old, 5 donors) who underwent circumcision or adult normal skin after plastic surgery (aged between 24 and 69 years old, 5 donors). Fetal epidermal keratinocytes were isolated from fetal skin of natural aborted fetus (~20 weeks, 2 donors). Skin from both genders were included. Collection was performed with patient and/or parental consent under an Institutional Review Board protocol approved by The First Affiliated Hospital of Sun Yat-sen University (reference no. 2022-350). In all instances, samples were obtained in de-identified manner and constituted non-human subjects research. The study was conducted in accordance with the Declaration of Helsinki principles. All ethical regulations relevant to human research participants were followed.

The skin’s subcutaneous tissue was excised using scissors and washed twice with Ca²^+^ and Mg²^+^-free PBS. The excised tissue was then incubated overnight at 4 °C in a 3 mg/mL Dispase solution (Sigma: D4693-1g). The following day, the epidermis was carefully separated from the dermis using forceps. The epidermal sheets were rinsed with PBS and then treated with 0.25% trypsin solution at 37 °C for 5–7 min before being neutralized with 10% fetal bovine serum (FBS). The resulting epidermal cells were filtered through a 70 µm cell strainer, centrifuged at 350 × *g* for 6 min, and the supernatant was discarded.

Primary fetal and adult keratinocytes were propagated in low calcium Epilife medium (Thermo Fisher Scientific: MEPI500CA) with recommended supplements to maintain an undifferentiated, stem and progenitor-enriched cell population. The same passage (passage 3 or 4) of fetal and adult keratinocytes were cultured in Epilife medium and characterized for epidermal stem cell markers, including CD49f, CD34, CD71, KRT14, KRT10, and ZFP42 before being used for all experiments.

### FACS analysis of surface markers

Passage 3 keratinocytes were resuspended using 100 µL staining buffer (PBS containing 3% BSA) at a concentration of 1 ×10^5^ cells/mL. The cells were labeled separately with FITC-conjugated CD49f (BioLegend: 313605), APC-conjugated CD71 (BioLegend: 334107), APC-conjugated CD34 (BioLegend: 128611), keratin 14 (Abcam: ab181595), keratin 10 (Abcam: ab76318) and ZFP42 (Invitrogen: 710190) primary antibodies or corresponding isotype controls. For keratin 14, keratin 10 and ZFP42 staining, goat anti-rabbit Alexa Fluor 488 (Invitrogen: A11034) was used as the secondary antibody. 5 μL of each antibody was added to the cell suspensions, followed by incubation at 4 °C for 1 h in the dark. After incubation, cells were centrifuged at 300 × *g* for 5 min, the supernatant was discarded, and the cells were washed twice with PBS containing 3% BSA. Finally, the cells were resuspended in PBS and analyzed using a CytoFlex flow cytometer (Beckman Coulter, USA). Data were analyzed using FlowJo v10.4 software (BD Biosciences).

### Cell culture and gene knockdown

Primary fetal and adult keratinocytes were propagated in low calcium Epilife medium (Thermo Fisher Scientific: MEPI500CA) with recommended supplements to maintain an undifferentiated, stem and progenitor enriched cell population. Same passage (passage 3 or 4) of fetal and adult keratinocytes were cultured in Epilife medium and used for all experiments. For synchronization, cells were treated with 20% FBS for 3 h. After that, cells were rinsed with PBS twice before changed back to the Epilife medium. Cells were harvested every 3 h within 24 h for rhythmic gene expression detection.

#### siRNA

siRNA targeting human *ZFP42, CRY1* (final concentration 20 nM) or control siRNA were transfected into keratinocytes using Lipofectamine RNAiMAX (Thermo Fisher Scientific: 13778150) reagent according to the manufacturer’s protocol and incubated for about 18 h. siRNA sequences were summarized in Supplementary Table 1.

#### shRNA

To knock down ZFP42 stably, lentiviruses expressing *ZFP42* shRNAs were used. shRNA retroviral constructs were generated by cloning oligos into the lentiviral vector pLKO.1. The lentiviral constructs (1 μg) were transfected using Lipofiter 3 (Hanbio, Shanghai, China) into 293 T cells, together with 1 μg of packaging plasmids pMD2.G (addgene: 12259) and psPAX2 (addgene: 12260). Viral supernatants were collected 48 h post-transfection and used to infect primary human keratinocytes. Cells were incubated in the viral supernatants and centrifuged at 1000 rpm for 1 h with hexadimethrine bromide (Sigma-Aldrich: H9268). Cells were transduced on two consecutive days. Cells were selected in puromycin 24 h after the last transduction to select for cells stably expressing the shRNAs (the lentiviral vector encodes a puromycin resistance gene). The shRNA sequence targeting the above genes is summarized in Supplementary Table 1.

### CCK8 assay and colony formation assays

Control or knockdown cells were seeded in 96-well plates (2000 cells per well) and measured using Cell Counting Kit-8 (CCK-8, Dojindo) according to the manufacturer’s instructions at different timepoints.

For clonogenic assays with primary human keratinocytes, 500 cells were plated in a 6-well plate and were grown for 10–14 days in Epilife medium and then fixed in 4% paraformaldehyde and stained with 0.1% crystal violet/0.1% rhodanile blue (Beyotime: C0121).

### Apoptosis assay and cell cycle assay

Control and ZFP42 or CRY1 knockdown cells were stained with Annexin V conjugated to Alexa Fluor 488 (Life Technologies: A13201) and analyzed using the Beckman flow cytometer (Beckman Coulter, USA) according to manufacturers’ instructions.

Control and ZFP42 or CRY1 knockdown cells were detached and washed with PBS containing 1% FBS, and then fixed with 80% ethanol at 4 °C for 15 min. Fixed cells were stained with 50 µg/mL propidium iodide in a PBS buffer containing 1% FBS, 0.05% Triton X-100, and 10 µg/mL RNaseA. After a 30-min incubation at room temperature, a flow cytometer was used to determine the percentage of cells in different phases of the cell cycle (G1, S, and G2/M phase) with CytoFLEX software (Beckman Coulter, CA).

### RNA isolation and RT-QPCR

Total RNA from cells was extracted using Trizol reagent (Thermo Fisher Scientific: 15596026) according to the manufacturer’s instructions. RNA quality and quantity were analyzed using Nanophotometer (Implen). One μg of total RNA was reversed transcribed using the PrimeScript™ RT Reagent Kit (Takara: RR047). cDNA was synthesized using the ABI Veriti 96-Well Thermal Cycler (Applied Biosystems). Quantitative PCR was carried out with TB Green® Premix Ex Taq™ II (Takara: RR820) on an Applied Biosystems™ QuantStudio™ 5 system. Gene expression was calculated using the ^ΔΔ^Ct method, with L32 used as internal control for normalization. The sequences of primers used in this study are provided in Supplementary Table 1.

### Immunostaining

Fetal or adult EPSCs were fixed in 4% paraformaldehyde for 11 min followed by blocking in PBS with 2.5% normal goat serum, 0.3% triton X-100, and 2% bovine serum albumin for 30 min. Primary antibodies used were Keratin 1 (Biolegend: 905204) at 1:200, MKI67 (Abcam: AB16667) at 1:200. The secondary antibodies used were Alexa 488 Goat anti-rabbit IgG (Thermo Fisher Scientific: A11034) at 1:500. Nuclear dye was stained with DAPI Fluoromount-G (Southern Biotech: 0100-20). Images were acquired using an Olympus/BX63 Upright Fluorescence Microscope.

### Western blot

Total protein was extracted using RIPA lysis buffer with protease inhibitors and quantified using BCA™ Protein Assay Kit. Fifty micrograms of protein from each sample were denatured and separated using BioRad Mini-PROTEAN Tetra System. The resolved proteins were transferred onto PVDF membranes (Milipore: IPVH00010) at 4 °C for two hours. Primary antibodies used include beta-actin (Santa Cruz: Sc-47778) at 1:30000, ZFP42 (Invitrogen: 710190) at 1:2000, CRY1 (Proteintech: 13474-1-AP) at 1:3000 overnight at 4 °C. Secondary antibodies including HRP conjugated anti-mouse IgG or anti-rabbit IgG (Cell Signaling Technology), were used at 1:2000.

Protein detection was performed using the enhanced chemiluminescence method (EpiZyme: SQ202) and analyzed with an Amersham ImageQuant 800 (Cytiva: 29399481).

### Construction of luciferase reporter plasmids

To construct the ZFP42 overexpression vector, the ZFP42 cDNA fragment was cloned into the BamHI (Thermo Fisher Scientific: FD0054) and EcoRI (Thermo Fisher Scientific: FD0274) sites of the pCDNA3.1(+) vector to generate the recombinant pCDNA3.1-ZFP42 plasmid. For the luciferase reporter plasmids containing the CRY1 or CCNB1 promoters, promoter regions of CRY1 (-2000 to -80 bp relative to the translation start site) and CCNB1 (−2000 to +7 bp relative to the translation start site) were amplified from genomic DNA of primary human EPSCs. The amplified sequences were inserted into the XhoI (Thermo Fisher Scientific: FD0694) and HindIII (Thermo Fisher Scientific: FD0504) cloning sites of the pGL4.10[luc2] basic luciferase vector (Promega: E6651).

### Dual-luciferase reporter assay

For the promoter activity assay, primary fetal EPSCs were seeded at a density of 50,000 cells per well in a 24-well plate. Cells were co-transfected with 1 μg of the pGL4.10[luc2] plasmid containing either the *CRY1* or *CCNB1* promoter, along with 1 μg of the pCDNA3.1-ZFP42 overexpression plasmid (or the pCDNA3.1-empty vector as a control), and 20 ng of pRL-SV40P plasmid (Addgene: 27163) as a normalization control. After 24 h of transfection, the culture medium was replaced with fresh medium. Forty-eight hours post-transfection, cells were harvested and lysed in luciferase lysis buffer and the luciferase activity was measured using the Dual-Luciferase Reporter Assay Kit (Vazyme: DL101-01) following the manufacturer’s instructions. Firefly and Renilla luciferase activities were quantified using the Dual-Luciferase Reporter Assay System (Promega). Firefly luciferase activity obtained from each sample was normalized to the Renilla luciferase activity from the same sample. Relative luciferase activity was calculated by comparing the normalized luciferase activity of knockdown or overexpressed cells to the control cells. Promoter sequences were summarized in Supplementary Table 1.

### RNA-Seq and bioinformatic analysis

Two biological replicates of both fetal and adult EPSCs were pooled to generate RNA-Seq data across circadian time points. RNA-Seq was performed using the DNBSEQ platform (BGI, China) with PE150. About 40 million pair-ended reads per sample were obtained. Reads were aligned to the GENCODE v19 transcriptome hg19 using Bowtie2 (v2.3.4.3) with default settings^60^. RSEM (v1.3.1) was used to generate gene and isoform expression levels and pheatmap (v1.0.8) was used to plot heatmap. Differential expression among samples was calculated using DESeq2^61^. Analysis of the read count distribution indicated that a threshold of ten reads per gene generally separated expressed from unexpressed genes, so all genes with fewer than ten reads were excluded from DESeq2 analysis. Gene lists for significantly upregulated or downregulated genes were created using *p* < 0.05 and 2-fold change. Enriched GO terms and KEGG analysis for RNA-seq differentially expressed gene sets were identified using Enrichr and Metascape^62–65^. For de novo promoter motif analysis, transcription factor motif finding was p erformed with motif finder program HOMER with the command *findMotifs.pl* on the interested genes. The motif with the most significant *p*-value predicted by HOMER was presented. To conduct motif analysis on non-rhythmic genes, we performed a matched case-control enrichment analysis: for each rhythmic gene in fetal EPSCs, we selected a matched set of non-rhythmic genes, ensuring they were similar in factors such as promoter length and GC content. We repeated this analysis several times to ensure robustness.

### Identification of rhythmic genes

To identify genes with a rhythmic expression from the fetal and adult EPSCs, the R package with Jonckheere–Terpstra–Kendall (JTK)_cycle (version:3.0) was used^30^. A *p* < 0.05 threshold is applied to identify rhythmic genes. The expression levels for all genes were normalized as TPM values and used for JTK analysis. DiffCircaPipeline (version:0.99.0) R package was used to detect different circadian characteristics^33^. Kronos/Cosinor (version:1.0.0) R package was used to produce the plots^66^. R package ClusterGVis (version: 0.0.6) was used for better clustering and visualizing the time series gene expression data^67^. Amplitude and phase estimations of oscillating genes were extracted from the DiffCircaPipeline algorithm.

### ChIP-QPCR

Five million control and CRY1 or ZFP42 knockdown cells and 3 μg of antibody were used for each antibody pulldown experiment for ChIP^14,19,68^. Cells for the CRY1 or ZFP42 pulldown ChIP-QPCR were fixed in both formaldehyde (1% final concentration, Thermo Fisher Scientific: 28908) and disuccinimidyl glutarate (2 mM final concentration, Thermo Fisher Scientific: 20593). As described before^18,19,69^, the cells were treated with Farnham lysis buffer and sheared with a syringe to assist lysis. After shearing, the cells were centrifuged and resuspended in SDS lysis buffer and sonicated using a Bioruptor® Plus system (Diagenode). Once the appropriate fragment size was achieved, the lysate was incubated with CRY1 (Proteintech: 13474-1-AP) or ZFP42 (Invitrogen: 710190) and Rabbit IgG (Millipore: 12-370). 50 μl of dynabeads™ Protein G (Invitrogen: 10004D) were added to each sample and rotated for 4 h at 4 °C. Wahses were then carried out to reduce non-specific binding. The beads were then placed in an elution buffer and the supernatant was isolated. Finally, the DNA was de-crosslinked and used for downstream ChIP-qPCR analysis. qPCR results are represented as enrichment of pulldown over IGG. Primers for ChIP-qPCR were summarized in Supplementary Table 1.

### Statistics and reproducibility

Results are expressed as mean±s.d. unless stated otherwise. Statistical comparisons between two groups were evaluated by unpaired Student’s *t* test, two-tailed. Statistical comparisons between three groups were evaluated by one-way ANOVA followed by Tukey’s multiple comparision. A probability (***p***) value of <0.05 was considered to indicate statistical significance.

### Reporting summary

Further information on research design is available in the Nature Portfolio Reporting Summary linked to this article.

## Supplementary information

Supplementary Information

Supplementary Data 1. List of circadian genes identified in fetal and adult EPSCs

Supplementary Data 2. List of circadian genes overlapped with previously published datasets

Supplementary Data 3. RNA-Seq datasets generated from ZFP42 knockdown and CRY1 knockdown experiments

Supplementary Data 4. source_data

Description of Additional Supplementary Files

Reporting Summary

## Data Availability

The raw RNA-Seq datasets generated from this study have been deposited in the Genome Sequence Archive at the National Genomics Data Center (https://ngdc.cncb.ac.cn/gsa-human/) with accession code HRA007097. CRY1 ChIP-Seq datasets were downloaded from Gene Expression Omnibus Repository (GEO) with the accession codes GSE130602^41^ and GSE230321^42^. The source data for the graphs in the study are found in Supplementary Data 4. Uncropped and unedited blot images are presented in Supplementary Fig. 7. Any other data that support the findings of this study, are available within the article, its Supplementary Information and from the corresponding author upon reasonable request.
