# Supplementary material for: ZFP42 maintains stemness and rhythmic transcription in human epidermal stem and progenitor cells via CRY1
Source: Commun Biol. 2026 Jan 21;9:291. doi: 10.1038/s42003-026-09576-0 (PMC12923524; doi:10.1038/s42003-026-09576-0)
Supplement: Supplementary file 1 — Supplementary Information [file 42003_2026_9576_MOESM1_ESM.pdf]

Supplementary Information

Supplementary Figures

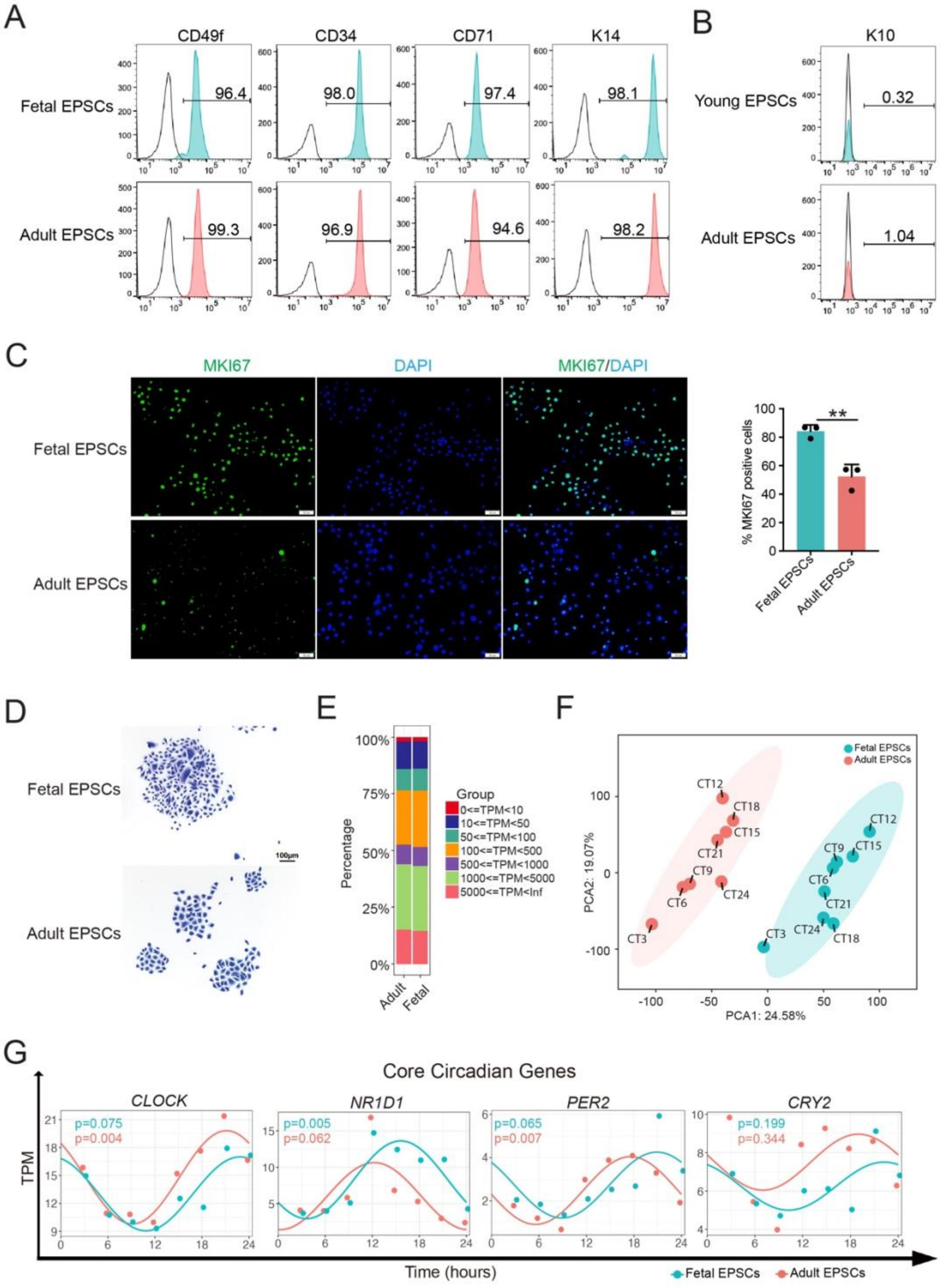

Supplementary Fig. 1. Characterization of fetal and adult EPSCs and their rhythmic gene expressions. A Flow cytometry analysis of cell surface markers CD49f, CD34, CD71, and Keratin 14 (K14). The data shown are the representative cell phenotype analyzed at passage 3. Blank peaks correspond to the isotype control and the color peaks to the fetal (teal) and adult (red) EPSCs with different antibodies of interest. Representative image is shown, N=3 independent experiments. **B** Flow cytometry analysis of Keratin 10 (K10) in young and adult EPSCs analyzed at passage 3. Blank peaks correspond to the isotype control. Representative image is shown, N=3 independent experiments. **C** Immunostaining of MKI67 in both fetal and adult EPSCs at passage 3 and quantification. Representative image is shown, N=3 independent experiment. **D** Clonogenic assay of both fetal and adult EPSCs. Representative image is shown, N=3 independent experiments. **E** Passage 3 fetal and adult EPSCs were synchronized with a short serum pulse and harvested every 3 hours within 24 hours, and the transcriptome was profiled respectively. Fractions of TPM values of average fetal and adult EPSCs expression levels in all timepoint samples. **F** PCA analysis of fetal (teal) and adult (red) samples from all timepoints. **G** Representative core circadian gene expression across 24 hours in fetal (teal) and adult EPSCs (red), including *CLOCK*, *NR1D1*, *PER2,* and *CRY2*. The Y-axis represents normalized expression for each gene. p-value was calculated using DiffCircaPipeline algorithm. CT, circadian time. TPM, Transcripts Per Million; PCA, Principal Component Analysis. ** p <0.01 (Student's t test). Mean values are shown with error bars representing standard deviations (SD).

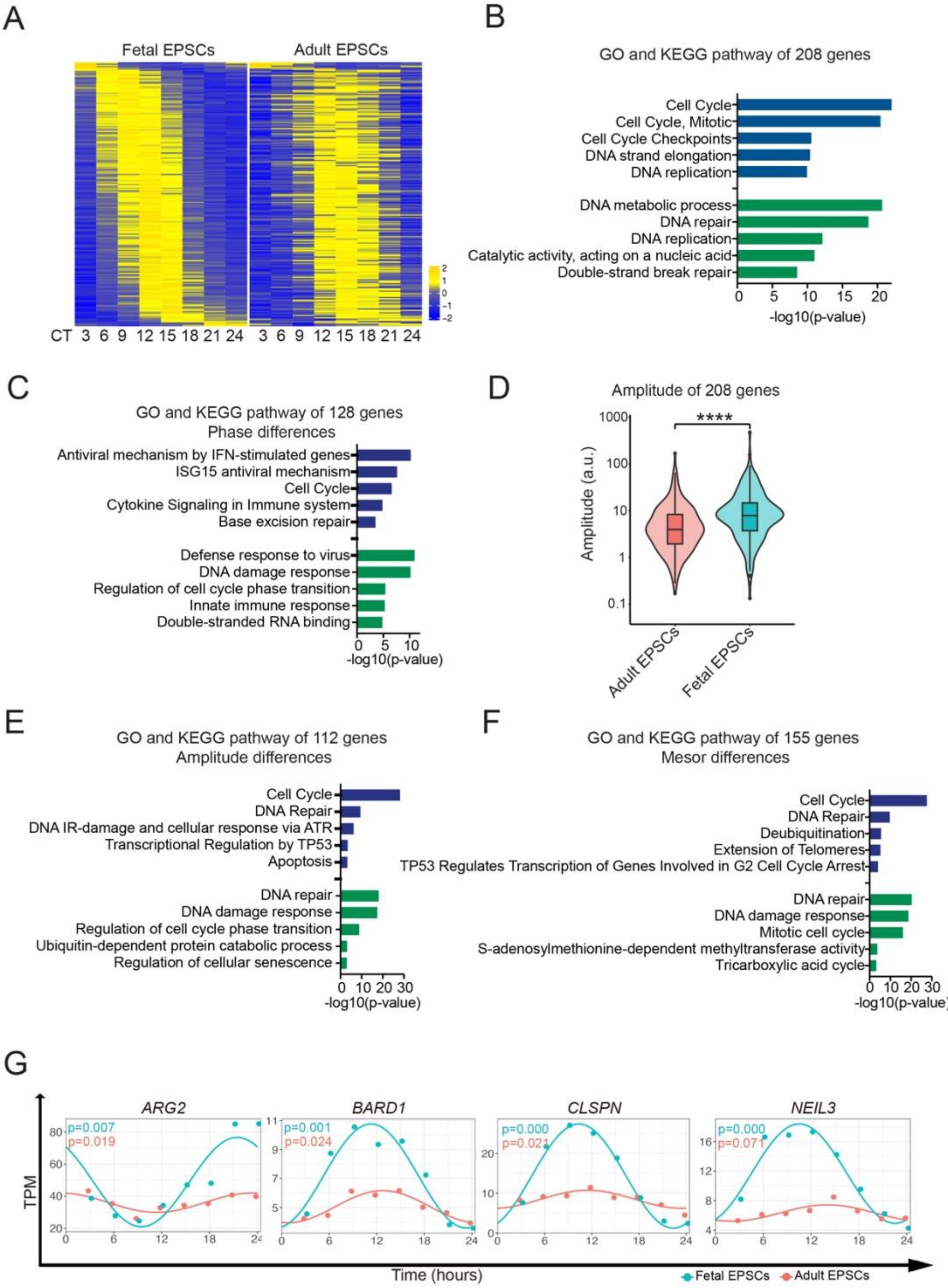
A
Fetal EPSCs
Adult EPSCs
CT 3 6 9 12 15 18 21 24 3 6 9 12 15 18 21 24
B
GO and KEGG pathway of 208 genes
Cell Cycle
Cell Cycle, Mitotic
Cell Cycle Checkpoints
DNA strand elongation
DNA replication
DNA metabolic process
DNA repair
DNA replication
Catalytic activity, acting on a nucleic acid
Double-strand break repair
-log10(p-value)
C
GO and KEGG pathway of 128 genes
Phase differences
Antiviral mechanism by IFN-stimulated genes
ISG15 antiviral mechanism
Cell Cycle
Cytokine Signaling in Immune system
Base excision repair
Defense response to virus
DNA damage response
Regulation of cell cycle phase transition
Innate immune response
Double-stranded RNA binding
-log10(p-value)
D
Amplitude of 208 genes
****
Amplitude (a.u.)
Adult EPSCs
Fetal EPSCs
E
GO and KEGG pathway of 112 genes
Amplitude differences
Cell Cycle
DNA Repair
DNA IR-damage and cellular response via ATR
Transcriptional Regulation by TP53
Apoptosis
DNA repair
DNA damage response
Regulation of cell cycle phase transition
Ubiquitin-dependent protein catabolic process
Regulation of cellular senescence
-log10(p-value)
F
GO and KEGG pathway of 155 genes
Mesor differences
Cell Cycle
DNA Repair
Deubiquitination
Extension of Telomeres
TP53 Regulates Transcription of Genes Involved in G2 Cell Cycle Arrest
DNA repair
DNA damage response
Mitotic cell cycle
S-adenosylmethionine-dependent methyltransferase activity
Tricarboxylic acid cycle
-log10(p-value)
G
ARG2
p=0.007
p=0.019
BARD1
p=0.001
p=0.024
CLSPN
p=0.000
p=0.021
NEIL3
p=0.000
p=0.071
TPM
Time (hours)
Fetal EPSCs
Adult EPSCs

Supplementary Fig. 2. Common rhythmic genes in fetal and adult EPSCs enriched cell cycle-associated pathways. A Heatmap shows expressions of 208 commonly rhythmic genes across time in fetal and adult EPSCs. **B** GO (Blue) and KEGG (Green) pathway analysis of the 208 rhythmic genes. **C** GO (Blue) and KEGG (Green) pathway analysis of 128 genes with phase differences among these 208 commonly rhythmic genes. **D** Amplitude analysis of the 208 common rhythmic genes. ****p <0.0001 (Student's t test). **E** GO (Blue) and KEGG (Green) pathway analyses of 112 genes are significantly different in amplitude among the 208 genes. **F** GO (Blue) and KEGG (Green) pathway analysis of 155 genes are significantly different in Mesor in the 208 common rhythmic genes. **G** Expression levels over time of representative rhythmic genes associated with cell senescence (*ARG2*), apoptosis (*BARD1*), DNA replication (*CLSPN*), and DNA repair (*NEIL3*) in fetal (teal) and adult (red) EPSCs. The Y-axis represents normalized expression for each gene. p-value was calculated using DiffCircaPipeline algorithm. GO, Gene Ontology. KEGG, Kyoto Encyclopedia of Genes and Genomes.

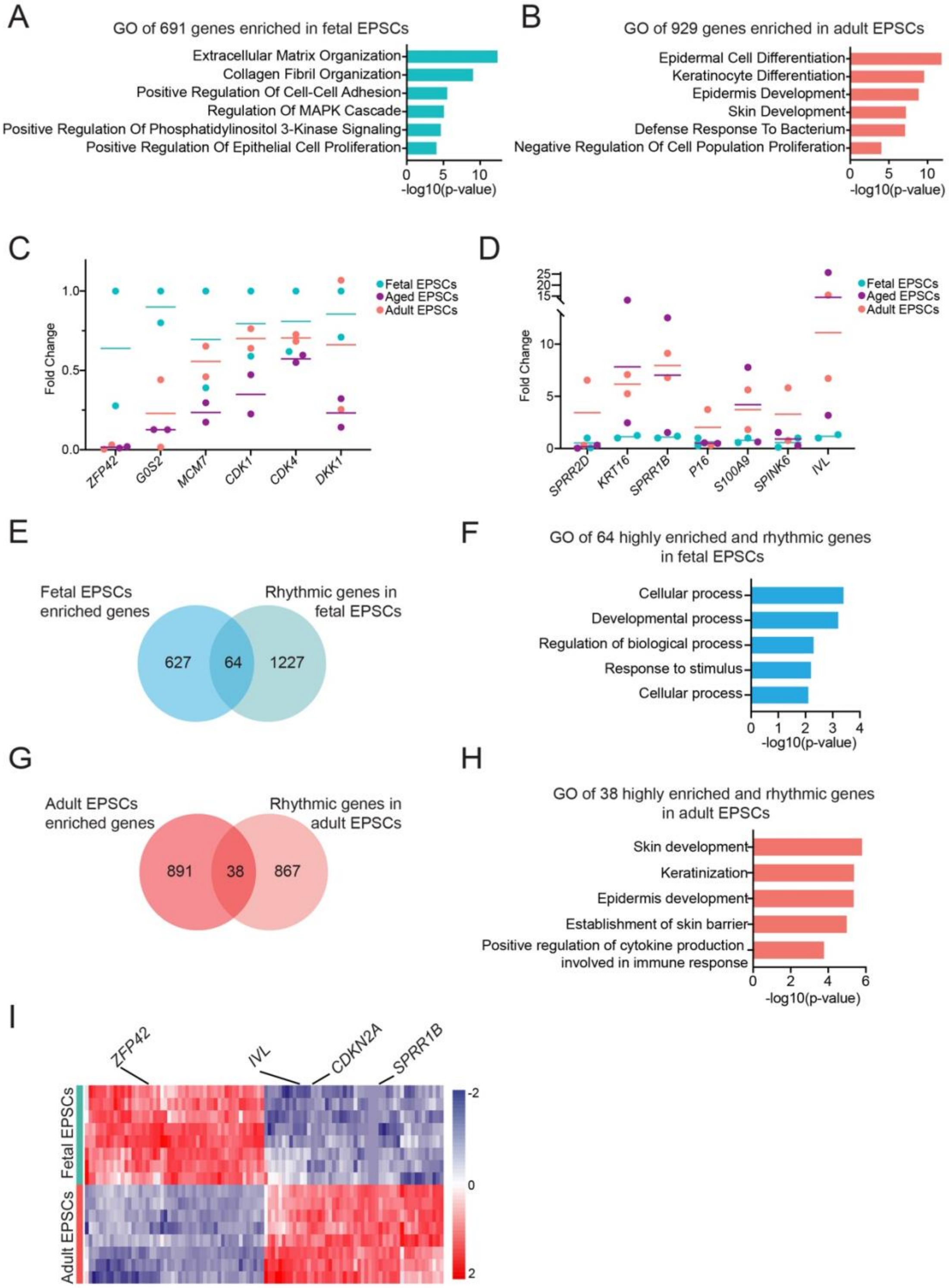
A
GO of 691 genes enriched in fetal EPSCs
Extracellular Matrix Organization
Collagen Fibril Organization
Positive Regulation Of Cell-Cell Adhesion
Regulation Of MAPK Cascade
Positive Regulation Of Phosphatidylinositol 3-Kinase Signaling
Positive Regulation Of Epithelial Cell Proliferation
-log10(p-value)
B
GO of 929 genes enriched in adult EPSCs
Epidermal Cell Differentiation
Keratinocyte Differentiation
Epidermis Development
Skin Development
Defense Response To Bacterium
Negative Regulation Of Cell Population Proliferation
-log10(p-value)
C
Fold Change
Fetal EPSCs
Aged EPSCs
Adult EPSCs
ZFP42
G0S2
MCM7
CDK1
CDK4
DKK1
D
Fold Change
SPRR2D
KRT16
SPRR1B
P16
S100A9
SPINK6
IVL
E
Fetal EPSCs enriched genes
Rhythmic genes in fetal EPSCs
627
64
1227
F
GO of 64 highly enriched and rhythmic genes in fetal EPSCs
Cellular process
Developmental process
Regulation of biological process
Response to stimulus
Cellular process
-log10(p-value)
G
Adult EPSCs enriched genes
Rhythmic genes in adult EPSCs
891
38
867
H
GO of 38 highly enriched and rhythmic genes in adult EPSCs
Skin development
Keratinization
Epidermis development
Establishment of skin barrier
Positive regulation of cytokine production involved in immune response
-log10(p-value)
I
ZFP42
IVL
CDKN2A
SPRR1B
Fetal EPSCs
Adult EPSCs

Supplementary Fig. 3. Differentially expressed genes in fetal and adult EPSCs. A GO analysis of 691 genes highly expressed in fetal EPSCs. **B** GO analysis of 929 genes highly expressed in adult EPSCs. **C & D** Expression of representative genes among fetal, young, and older adult EPSCs from two biological replicates measured by RT-QPCR. **E** Venn diagram of the fetal rhythmic genes (data from **Fig. 1A**) and highly expressed genes in fetal EPSCs. **F** GO analysis of the 64 highly enriched and rhythmic genes in fetal EPSCs. **G** Venn diagram of the adult rhythmic genes (data from **Fig. 1A**) and highly expressed genes in adult EPSCs. **H** GO analysis of 38 highly enriched and rhythmic genes in adult EPSCs. **I** Heatmap of the top 100 DEGs between fetal and adult EPSCs. GO, Gene Ontology; RT-QPCR, reverse transcription quantitative PCR.

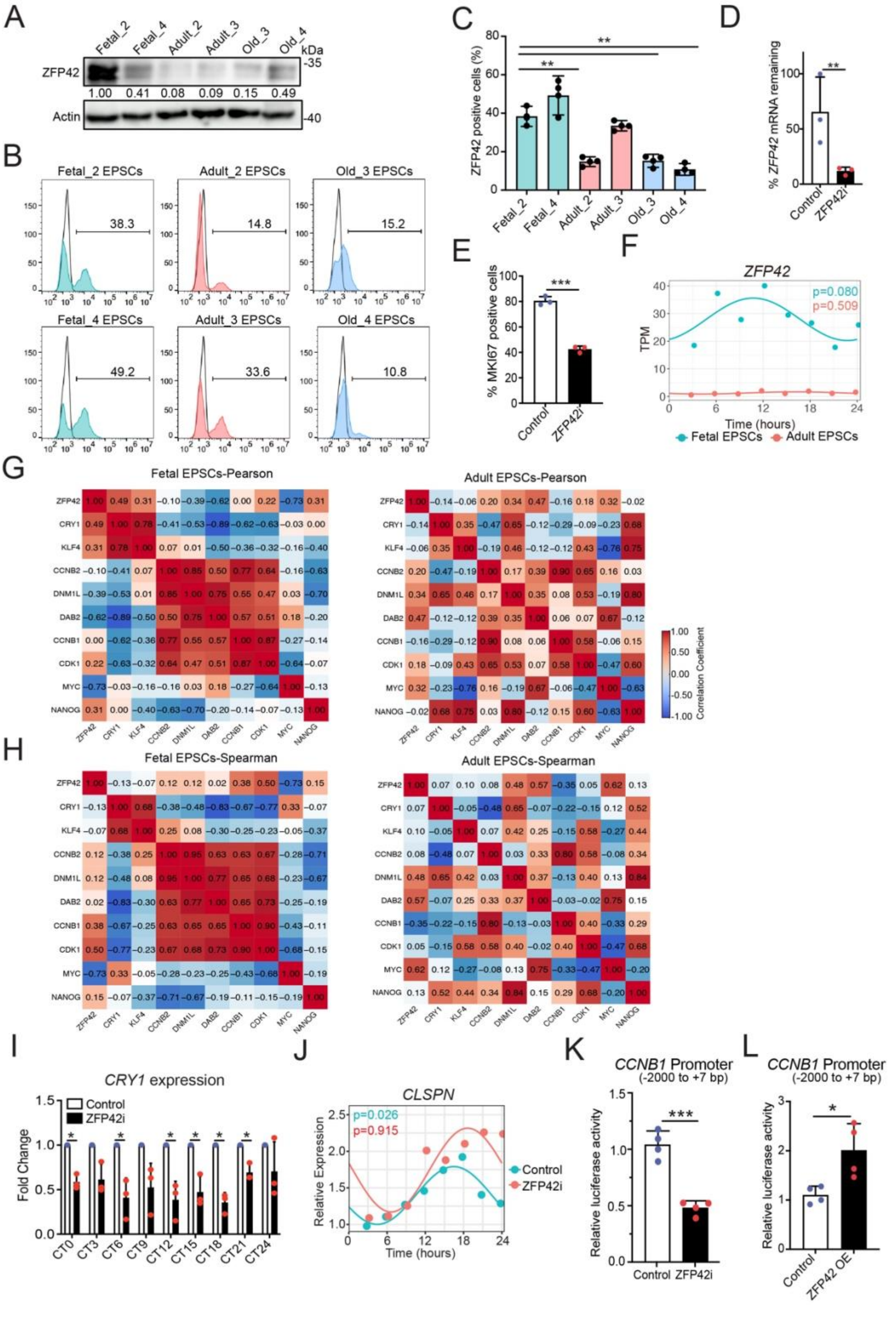
A
Fetal_2
Fetal_4
Adult_2
Adult_3
Old_3
Old_4
kDa
ZFP42
-35
1.00 0.41 0.08 0.09 0.15 0.49
Actin
-40
B
Fetal_2 EPSCs 38.3
Adult_2 EPSCs 14.8
Old_3 EPSCs 15.2
Fetal_4 EPSCs 49.2
Adult_3 EPSCs 33.6
Old_4 EPSCs 10.8
C
ZFP42 positive cells (%)
D
% ZFP42 mRNA remaining
Control
ZFP42i
E
% MKI67 positive cells
F
ZFP42
p=0.080
p=0.509
TPM
Time (hours)
Fetal EPSCs
Adult EPSCs
G
Fetal EPSCs-Pearson
Adult EPSCs-Pearson
Correlation Coefficient
ZFP42
CRY1
KLF4
CCNB2
DNM1L
DAB2
CCNB1
CDK1
MYC
NANOG
H
Fetal EPSCs-Spearman
Adult EPSCs-Spearman
I
CRY1 expression
Control
ZFP42i
Fold Change
CT0 CT3 CT6 CT9 CT12 CT15 CT18 CT21 CT24
J
CLSPN
p=0.026
p=0.915
Relative Expression
Time (hours)
Control
ZFP42i
K
CCNB1 Promoter
(-2000 to +7 bp)
Relative luciferase activity
Control
ZFP42i
L
CCNB1 Promoter
(-2000 to +7 bp)
Relative luciferase activity
Control
ZFP42 OE

Supplementary Fig. 4. ZFP42 maintains self-renewal and inhibits differentiation in human EPSCs. **A** Measurement of ZFP42 expression at protein level in fetal, adult and old EPSCs (n=2 donors). **B** Flow cytometry analysis of ZFP42 in fetal, adult, and old EPSCs. **C** Quantification of ZFP42-positive cells in fetal, adult and old EPSCs derived from single donors (n=3 independent experiments). **D** Measurement of *ZFP42* expression at mRNA level in Control (n=3) and ZFP42 knockdown (ZFP42i) (n=3) young EPSCs using siRNAs as measured by RT-QPCR at 3 days post-transfection. **E** Quantification of Mki67 positive cells in Control (n=3) and ZFP42i young EPSCs (n=3) as measured by immunostaining. **F** ZFP42 expression over time in fetal and adult EPSCs. $\boldsymbol{p}$-value was calculated using DiffCircaPipeline algorithm. **G & H** Pearson and Spearman correlation and clustering of ZFP42 and other genes over the time course in fetal and adult EPSCs. **I** qPCR analysis of *CRY1* expression upon ZFP42 knockdown in young EPSCs over time. N=3 independent experiments. **J** *CLSPN* expression over time in Control and ZFP42i young EPSCs as measured by RT-QPCR. **K** Measurement of *CCNB1* promoter activity in both Control (n=4) and ZFP42i fetal EPSCs (n=4) using a dual-luciferase reporter assay. Relative luciferase activity was calculated by comparing the normalized luciferase activity of knockdown cells to the control cells. **L** Measurement of *CCNB1* promoter activity in both Control (n=4) and ZFP42 overexpression fetal EPSCs (n=4) using a dual-luciferase reporter assay. Relative luciferase activity was calculated by comparing the normalized luciferase activity of overexpressed cells to the control cells. N=3 independent experiments. *$\boldsymbol{p}$ <0.05, ** $\boldsymbol{p}$ <0.01, ***$\boldsymbol{p}$ <0.001 (Student's t test). RT-QPCR, reverse transcription quantitative PCR. OE, overexpression. Mean values are shown with error bars representing standard deviations (SD).

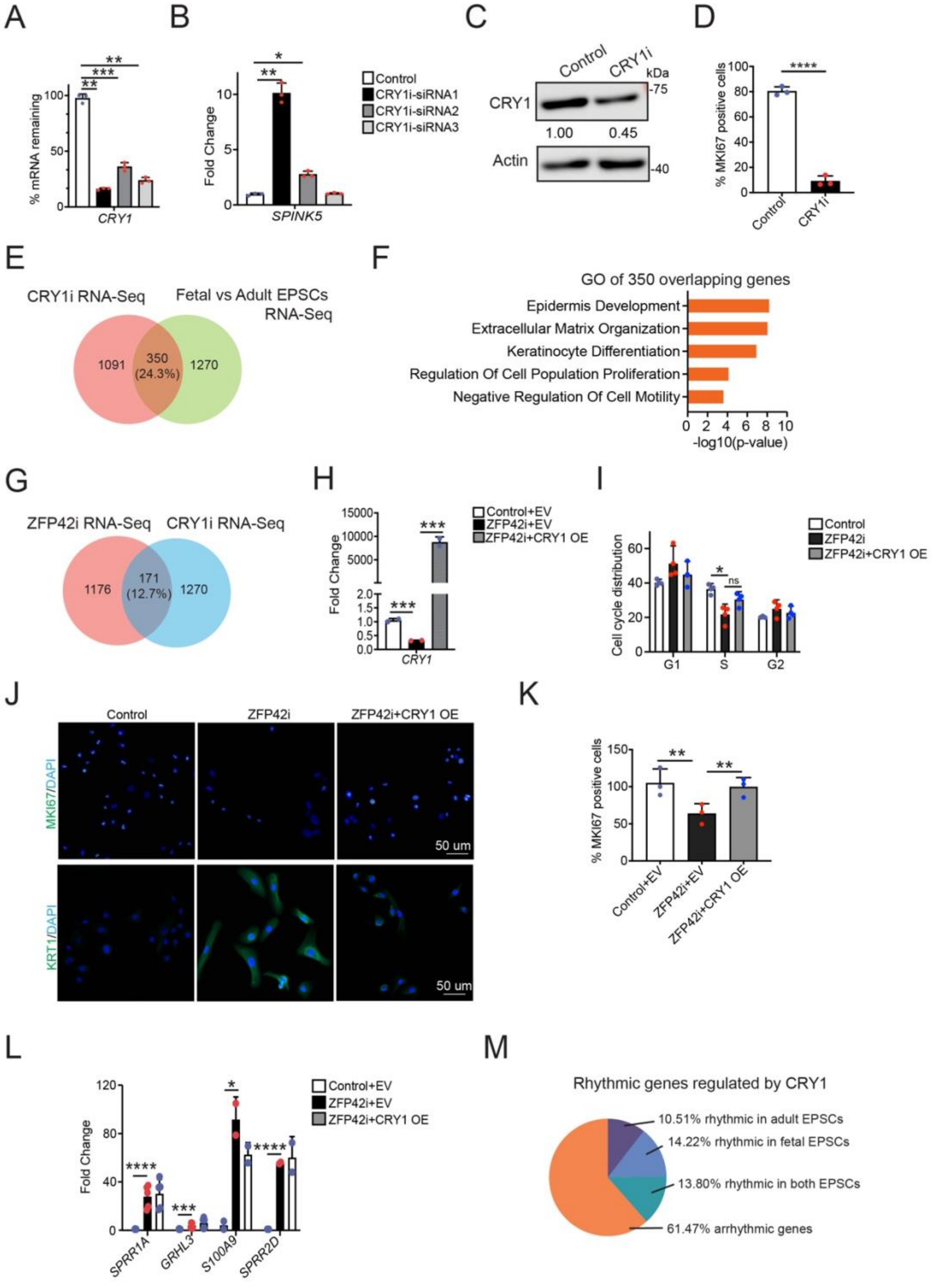

A
B
C
D
% mRNA remaining
CRY1
Fold Change
SPINK5
Control
CRY1i-siRNA1
CRY1i-siRNA2
CRY1i-siRNA3
Control
CRY1i
kDa
CRY1
-75
1.00
0.45
Actin
-40
% MKI67 positive cells
Control
CRY1i
E
CRY1i RNA-Seq
Fetal vs Adult EPSCs RNA-Seq
1091
350 (24.3%)
1270
F
GO of 350 overlapping genes
Epidermis Development
Extracellular Matrix Organization
Keratinocyte Differentiation
Regulation Of Cell Population Proliferation
Negative Regulation Of Cell Motility
-log10(p-value)
G
ZFP42i RNA-Seq
CRY1i RNA-Seq
1176
171 (12.7%)
1270
H
Fold Change
CRY1
Control+EV
ZFP42i+EV
ZFP42i+CRY1 OE
I
Cell cycle distribution
G1
S
G2
ns
Control
ZFP42i
ZFP42i+CRY1 OE
J
Control
ZFP42i
ZFP42i+CRY1 OE
MKI67/DAPI
KRT1/DAPI
50 um
50 um
K
% MKI67 positive cells
Control+EV
ZFP42i+EV
ZFP42i+CRY1 OE
L
Fold Change
SPRR1A
GRHL3
S100A9
SPRR2D
Control+EV
ZFP42i+EV
ZFP42i+CRY1 OE
M
Rhythmic genes regulated by CRY1
10.51% rhythmic in adult EPSCs
14.22% rhythmic in fetal EPSCs
13.80% rhythmic in both EPSCs
61.47% arrhythmic genes

Supplementary Fig. 5. CRY1 overexpression rescues the decreased proliferation in human EPSCs resulted by ZFP42 knockdown. A Proliferating human EPSCs derived from young kid were knocked down with Control (n=3) or three independent CRY1 siRNAs (n=3) and the remaining *CRY1* mRNA levels were measured by RT-QPCR. **B** Measurement of epidermal differentiation marker *SPINK5* mRNA levels in Control (n=3) and CRY1 knockdown (CRY1i) young EPSCs (n=3). **C** Measurement of CRY1 at protein level after knockdown in young EPSCs using western blot. Representative image is shown, N=3 independent experiments. **D** Quantification of Mki67 positive cells in Control (n=3) and CRY1i young EPSCs (n=3) 3 days after transfection as measured by immunostaining. **E** Venn diagram of DEGs upon CRY1 knockdown and DEGs between fetal and adult EPSCs. **F** GO terms of the 350 genes that overlapped between CRY1i RNA-Seq and DEGs of fetal and adult EPSCs. **G** Venn diagram of DEGs upon ZFP42 and CRY1 knockdown. **H** Measurement of gene expression upon ZFP42 knockdown or ZFP42 knockdown with CRY1 OE by RT-QPCR. EV is empty vector and CRY1 OE is overexpression of CRY1. N=3 independent experiments. **I** Cell cycle analysis of Control, ZFP42i, and ZFP42i+CRY1 OE young EPSCs. **J** Immunostaining of MKI67 and KRT1 in Control, ZFP42i, ZFP42i+CRY1 OE young EPSCs. **K** Quantification of MKI67-positive cells from Supplementary Figure 5J. **L** Measurement of differentiation-associated genes (*SPRR1A, GRHL3, S100A9,* and *SPRR2D*) expression upon ZFP42 knockdown or ZFP42 knockdown with CRY1 OE young EPSCs by RT-QPCR. N=3 independent experiments. **M** Analysis of rhythmic genes in fetal and adult EPSCs (data in **Fig. 1A**) regulated by CRY1 (RNA-Seq). *p <0.05, ** p <0.01, ***p <0.001 (Student's t test was performed for comparision between two groups and one-way ANOVA followed by Tukey’s multiple comparison for 3 groups). n.s. not significant. RT-QPCR, reverse transcription quantitative PCR, DEG, differentially expressed genes. EV, empty vector. OE, overexpression. Mean values are shown with error bars representing standard deviations (SD)

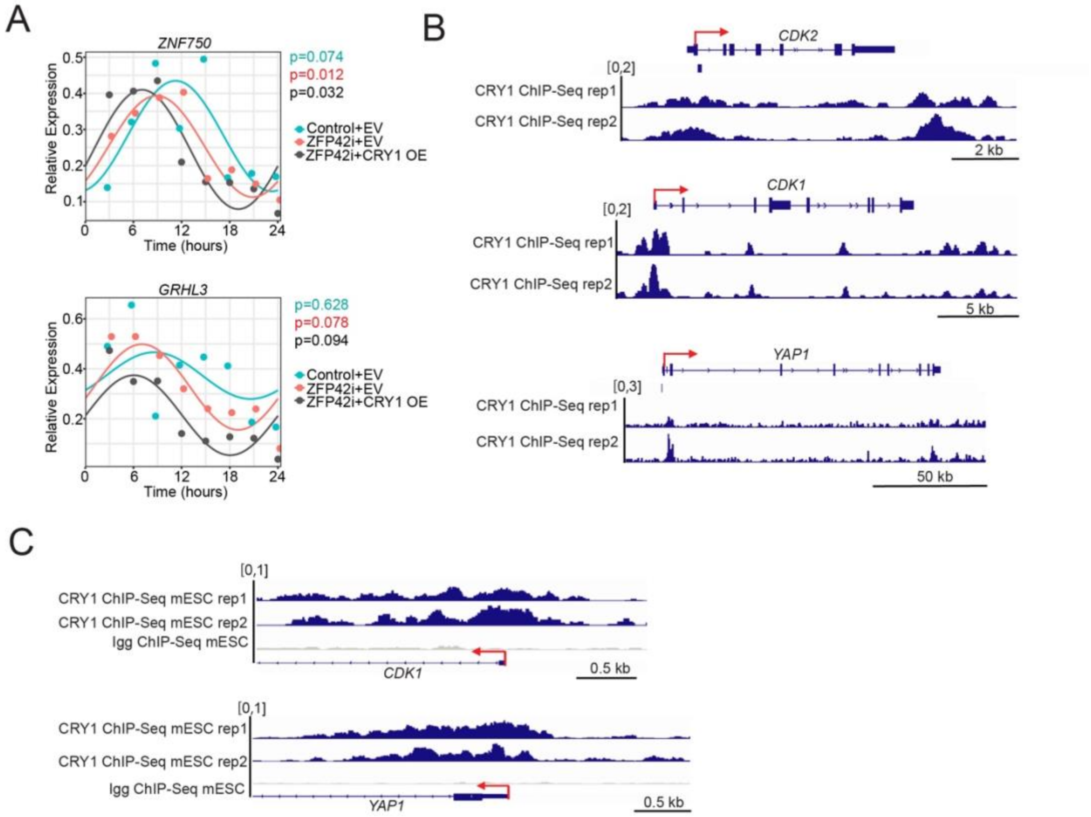
A
ZNF750
p=0.074
p=0.012
p=0.032
Relative Expression
Time (hours)
Control+EV
ZFP42i+EV
ZFP42i+CRY1 OE
GRHL3
p=0.628
p=0.078
p=0.094
B
CDK2
CRY1 ChIP-Seq rep1
CRY1 ChIP-Seq rep2
2 kb
CDK1
5 kb
YAP1
50 kb
C
CRY1 ChIP-Seq mESC rep1
CRY1 ChIP-Seq mESC rep2
Igg ChIP-Seq mESC
CDK1
0.5 kb
YAP1
0.5 kb

Supplementary Fig. 6. CRY1 fails to restore the rhythmic expression of differentiation genes in human EPSCs following ZFP42 loss. A Expressions of *ZNF750* and *GRHL3* over time in Control+EV, ZFP42i+EV, and ZFP42i+CRY1 OE young EPSCs. **B** Gene tracks showing CRY1 binding to *CDK1*, *CDK2,* and *YAP1* genomic regions. CRY1 ChIP-Seq is shown in dark blue. Y-axis shows RPM and X-axis shows position along the gene. **C** Gene tracks of *CDK1* and *YAP1*. CRY1 ChIP-Seq in mouse ESCs is shown in dark blue. Igg is shown in grey. Y-axis shows RPM and X-axis shows position along gene. EV, empty vector. OE, overexpression.

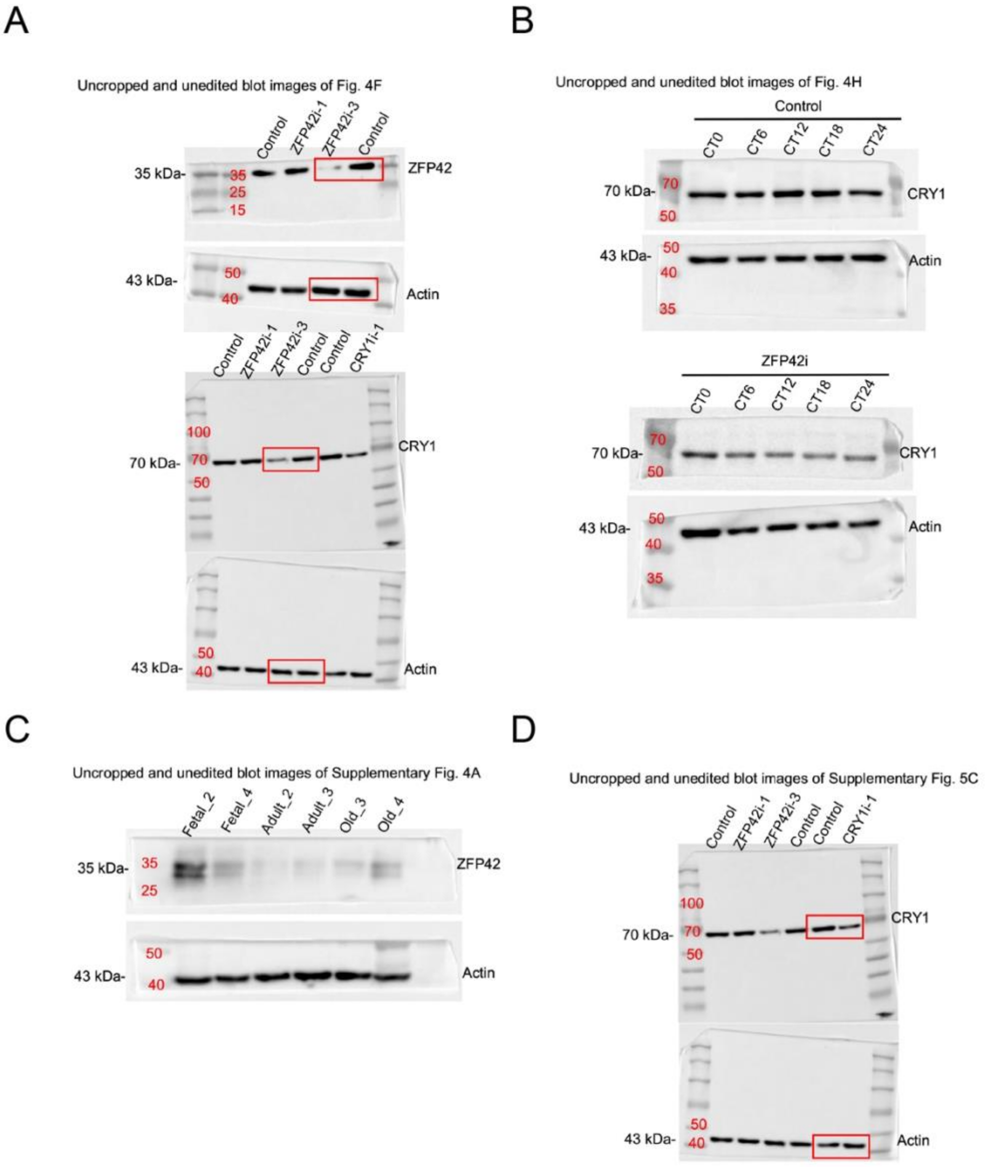

Supplementary Fig. 7. Uncropped and unedited blot images in Figure 4F, 4H, and Supplementary Fig. 4A and 5C.

Supplementary Table 1. Primer sequences and siRNA shRNA oligonucleotides used in this study.

RT-qPCR Primers	Forward	Reverse
ZFP42	GTGGGAAAGCGTTCGTTG	GCAGCCTTGAAAGGGACA
FGFBP1	TCTGGGCAACACCCAGAT	GGCATGAGGTTGGATTGC
KRT15	AGATCGCTACTTACCGCAGC	CCACCTGTCCATCCACTGAC
S100A9	CGCAGCTGGAACGCAACAT	GCCCTCGTCACCCTCGTG
CYR61	GGTTTCCAGGGCACACCT	AGTGTCCATCCGCACCAG
MYC	CGGACACCGAGGAGAATG	GCTTGGACGGACAGGATG
BMAL1	CCCTGGGCCATCTCGATTAT	TCATCCAGCCCCATCTTTGT
CLOCK	TGGGTTGGTGGAAGAAGATGA	CCAGGAAGCATGGATCCCA
PER1	CGCGTCATGATGACCTACCA	GCTGGTCCTCAGAAAACCGA
PER2	CTGGCCATCCACAAAAAGAT	CCTCCCAATGATGAAGGAGA
CRY1	TGGCAACTGAAGCTGGAGT	CTCCACCTGGCCACACTG
CRY2	GACCCACACATGGTTCCTACT	TCCAAGTGCTTATCCAGGCG
NR1D1	GGCTTCTCCCAGTTTCCACA	GTGTTGTTGTCATTGGGGGC
NR1D2	TGCTGTTCGGTTTGGTCGTA	TGGCAAGGCTGTCTGTTCAT
PRC1	GCGAAATTCTTTGTATGCCCCA	CCTCACGCCTAGAAGCCTTT
BUB1	AGACATTATATGAAAAGGAAAGAAGCA	GTCTCCACCACCTGATGCAA
MKi67	TGGCCAAGAACGCCTAAG	GGCCATTGCTTTGTGCTT
GRHL3	GCCAGTTCTACCCCGTCA	GTCAATGACCCGCTGCTT
KRTDAP	AATTATGCGTCACGACCCGA	TCCCAGTTGAGGAAAGGAAGT
SCEL	TCAGAAACAATCAGAGCCAAGAC	TCCAGTGTTAGAGCTTCCATTCT
FLG	GGCAAATCCTGAAGAATCCA	TGCTTTCTGTGCTTGTGTCC
GOS2	GTGGTGCTCGGCCTGATG	TGTCCTGCTGCTTGCCTTT
MCM7	CTCAGGAGTGGGGCTTACGG	TGTCGAACTCATCAATGCAGC
CDK1	GCTGGGGTCAGCTCGTTA	TTCCACTTCTGGCCACACT
CDK2	TGGCGCTTCATGGAGAACTT	CATCCAGCAGCGTGTCCAG
CDK4	GCTGCCTCCAGAGGATGA	GCTGCAGAGCTCGAAAGG
DKK1	TCCAACGCTATCAAGAACCTG	CAGGCGAGACAGATTTGCAC
SPRR2D	GAGAACCTGGTGAGTATCCTG	GCTCTTGGGTGGACACTTTG
KRT16	CGCAGCCCATTTTGCAGATT	GTCGGCCTCCACAGTCTG
P16	CTTCCTGGACACGCTGGT	ATGCGGGCATGGTTACTG
S100A9	CGCAGCTGGAACGCAACAT	GCCCTCGTCACCCTCGTG
SPINK6	GCTCCTCTCTCTGGCTCTTTTC	GGTTAGATTCCCGAGTGCAGTA
IVL	AAAGCACCTAGAGCACCC	GGTTGAATGTCTTGGACCT
SPP1	GTCCAACGAAAGCCATGACC	TCTACATCATCAGAGTCGTTCGA
SPRR1B	CCATGCATCCCCAAAACCAA	GCTGGTGCTGGAGTGACTAT
SPRR1A	GCCACTGGATACTGAACA	AGGAAGACTAGGGATGGTT
DSC1	AGACTCCCCATGCAGACATC	GTCCACCTCCTTTGTTGGAA
SPINK5	ACCTGTAGGCGACTTGCATC	TGGCACATTTCCTGATCTTCA
CCND1	GCTGCGAAGTGGAAACCATC	CCTCCTTCTGCACACATTTGAA
CCNE1	ACTCAACGTGCAAGCCTCG	GCTCAAGAAAGTGCTGATCCC
CDKN1B	TAATTGGGGCTCCGGCTAACT	TGCAGGTCGCTTCCTTATTCC
YAP	AACGCCGTCATGAACCCCA	GTCAGTGTCCCAGGAGAAACA

ChIP-qPCR primers	Forward	Reverse
PER1	CCACTAAGGTCAGGGCTGTG	GATGTTGTGTTCTGCAAGGCT
CDK1	TGAGTATAATAAATTTGAACTGTGCCA	GCTCTCCTCCAGTCGGGA
CRY1	GACGGCCCCAGGAGATTC	CCTCGACCACCGCCTCTA
CDK2	CGCTTAAGAAAATCCGCCTGG	CTTCACCTTCTACTGGCCCC
YAP1	GCGTTTGAGGCGAGTTTCTG	CTCGTTGCCTTTCCCCGC

siRNA sequences	
ZFP42-1	GCAAGGCAAGUCAAGCCAATT
ZFP42-2	GCUCCCUUGAAUGUUCUUUTT
ZFP42-3	GCGCUUCUCUCUGGACUUUTT
CRY1-1	GAUGCAGAUUGGAGCAUAAAUdTdT
CRY1-2	ACAAGAUCAUAGAACUCAAUGdTdT
CRY1-3	CAGCAGCUUUCACGAUAUAGAdTdT

shRNA sequences		
ZFP42-J1	CCGGGTTGGAGAGAATTCGCTTGCTCGAGCAAG CGAATTCTCTCCAACTTTTTGAATT	
ZFP42-K2	CCGGGAAGAGTGATCAGTGACAACTCGAGTTG TCACTGATCACTCTTCTTTTTGAATT	
ZFP42-H1	CCGGGGATGATTTCTCAGACTGTCTCGAGACAG TCTGAGAAATCATCCTTTTTGAATT	

Plasmid sequences	Forward	Reverse
CRY1_promoter	CCTGAGCTCGCTAGCCTCGAGCAGATTGGCTCT CTAGAGTATTGAATTT	CAGTACCGGATTGCCAAGCTTCCTC ACGTTTCTGAAGTGTGTTTAC
CCNB1_promoter	CCTGAGCTCGCTAGCCTCGAGTGCATATTTGAG TAAAAGAAACCATTC	CAGTACCGGATTGCCAAGCTTGAGA AGAGCCAGCCTAGCCTC
ZFP42_CDS	CTAGCGTTTAAACTTAAGCTTATGAGCCAGCAA CTGAAGAAACG	CTAGCGTTTAAACTTAAGCTTATGA GCCAGCAACTGAAGAAACG
