# Supplementary material for: ZFP42 maintains stemness and rhythmic transcription in human epidermal stem and progenitor cells via CRY1
Source: Commun Biol. 2026 Jan 21;9:291. doi: 10.1038/s42003-026-09576-0 (PMC12923524; doi:10.1038/s42003-026-09576-0)
Supplement: Supplementary file 6 — Description of Additional Supplementary Files [file 42003_2026_9576_MOESM6_ESM.pdf]

Description of Additional Supplementary Files

File name: Supplementary Data 1

Description: List of circadian genes identified in fetal and adult EPSCs

File name: Supplementary Data 2

Description: List of circadian genes overlapped with previously published datasets

File name: Supplementary Data 3

Description: RNA-Seq datasets generated from ZFP42 knockdown and CRY1 knockdown experiments

File name: Supplementary Data 4

Description: Source data
