# Supplementary material for: ZFP42 maintains stemness and rhythmic transcription in human epidermal stem and progenitor cells via CRY1
Source: Commun Biol. 2026 Jan 21;9:291. doi: 10.1038/s42003-026-09576-0 (PMC12923524; doi:10.1038/s42003-026-09576-0)
Supplement: Supplementary file 7 — Reporting Summary [file 42003_2026_9576_MOESM7_ESM.pdf]

nature portfolio

Corresponding author(s): Jingting Li

Last updated by author(s): Jan 8, 2026

Reporting Summary

Nature Portfolio wishes to improve the reproducibility of the work that we publish. This form provides structure for consistency and transparency in reporting. For further information on Nature Portfolio policies, see our Editorial Policies and the Editorial Policy Checklist.

Statistics

For all statistical analyses, confirm that the following items are present in the figure legend, table legend, main text, or Methods section.

n/a	Confirmed	
☐	☒	The exact sample size (*n*) for each experimental group/condition, given as a discrete number and unit of measurement
☐	☒	A statement on whether measurements were taken from distinct samples or whether the same sample was measured repeatedly
☐	☒	The statistical test(s) used AND whether they are one- or two-sided<br>*Only common tests should be described solely by name; describe more complex techniques in the Methods section.*
☒	☐	A description of all covariates tested
☐	☒	A description of any assumptions or corrections, such as tests of normality and adjustment for multiple comparisons
☐	☒	A full description of the statistical parameters including central tendency (e.g. means) or other basic estimates (e.g. regression coefficient) AND variation (e.g. standard deviation) or associated estimates of uncertainty (e.g. confidence intervals)
☐	☒	For null hypothesis testing, the test statistic (e.g. *F*, *t*, *r*) with confidence intervals, effect sizes, degrees of freedom and *P* value noted<br>*Give P values as exact values whenever suitable.*
☐	☒	For Bayesian analysis, information on the choice of priors and Markov chain Monte Carlo settings
☐	☒	For hierarchical and complex designs, identification of the appropriate level for tests and full reporting of outcomes
☐	☒	Estimates of effect sizes (e.g. Cohen's *d*, Pearson's *r*), indicating how they were calculated

Our web collection on statistics for biologists contains articles on many of the points above.

Software and code

Policy information about availability of computer code

Data collection	Rhythmic gene detection was performed using the JTK_CYCLE R package (Hughes et al., 2010), a nonparametric algorithm designed for identifying rhythmic components in genome-scale data. To compare circadian parameters between fetal and adult epidermal stem cells, the DiffCircaPipeline R package (Xue et al., 2023) was used, which enables multifaceted characterization of differential rhythmicity. All analyses were conducted using R version 4.4.2.
Data analysis	Bar plots were drawn using GraphPad Prism V10;<br>RNA-seq reads were aligned to the reference genome using Bowtie2 (Langmead and Salzberg, 2012), a fast and memory-efficient aligner suitable for short-read alignment. All alignments were performed using default parameters unless otherwise specified.Enrichr (Kuleshov, M.V., Jones, M.R., Rouillard, A.D., Fernandez, N.F., Duan, Q., Wang, Z., Koplev, S., Jenkins, S.L., Jagodnik, K.M., Lachmann, A., et al. (2016).<br>Gene set enrichment analysis was performed using Enrichr, an interactive and comprehensive web-based tool for gene set knowledge discovery (Xie et al., 2021). The tool provides access to a wide range of gene set libraries and statistical models for enrichment calculation.<br>For transcriptomic data correlation analysis, deepTools (Ramírez et al., 2014) was used to calculate pairwise Pearson correlation coefficients and generate correlation heatmaps using the plotCorrelation function. Default parameters were used unless otherwise specified.<br>De novo motif analysis was performed using HOMER (Hypergeometric Optimization of Motif EnRichment), a suite of tools for finding and annotating enriched motifs in genomic regions (Heinz et al., 2010). The default settings were used unless otherwise specified.

For manuscripts utilizing custom algorithms or software that are central to the research but not yet described in published literature, software must be made available to editors and reviewers. We strongly encourage code deposition in a community repository (e.g. GitHub). See the Nature Portfolio guidelines for submitting code & software for further information.

Data

Policy information about availability of data

All manuscripts must include a data availability statement. This statement should provide the following information, where applicable:

- Accession codes, unique identifiers, or web links for publicly available datasets
- A description of any restrictions on data availability
- For clinical datasets or third party data, please ensure that the statement adheres to our policy

The RNA-Seq datasets generated from this study has been deposited in China Genomics Data Center with GSA (HRA007097). CRY1 ChIP-Seq datasets was downloaded from GEO database (GSE130602 and GSE230321).

Research involving human participants, their data, or biological material

Policy information about studies with human participants or human data. See also policy information about sex, gender (identity/presentation), and sexual orientation and race, ethnicity and racism.

Reporting on sex and gender	This study investigates fundamental mechanisms using human epidermal stem cells. The research question is not sex- or gender-specific, and sex and gender were not considered in the study design. Donor sex/gender information was not collected, and no sex- or gender-based analyses were performed. The results are intended to reflect general biological mechanisms and are not specific to any particular sex or gender.
Reporting on race, ethnicity, or other socially relevant groupings	Human foreskin tissues were obtained from de-identified donors undergoing circumcision at the First Affiliated Hospital of Sun Yat-sen University. Sample collection was conducted under approved institutional protocols (Ethics Approval No. IIT-2022-039), with written informed consent obtained from all participants or their legal guardians.<br>No personally identifiable or socially constructed information (such as gender identity, ethnicity, or socioeconomic status) was collected or analyzed. This study focused exclusively on basic cellular and molecular mechanisms, and thus did not consider social, demographic, or gender-related variables.
Population characteristics	Human epidermal stem cells were isolated from foreskin tissues obtained from de-identified donors undergoing circumcision at the First Affiliated Hospital of Sun Yat-sen University. Skin tissues from back or breast were collected from other donors. Donors included fetal, pediatric, adolescent, and elderly individuals. However, detailed individual-level information such as exact age, phenotypic characteristics, medical history, or treatment categories was not collected. The study focused on basic mechanistic investigations at the cellular level, and no covariate-relevant participant characteristics were included or analyzed.
Recruitment	Donors were patients undergoing circumcisio, plastic surgery, or abortions at the First Affiliated Hospital of Sun Yat-sen University. Samples were collected with informed consent during routine clinical procedures. As samples were collected from discarded tissue without active recruitment, the risk of self-selection or other biases is minimal and unlikely to affect the study's mechanistic conclusions.
Ethics oversight	The First Affiliated Hospital of Sun Yat-sen University

Note that full information on the approval of the study protocol must also be provided in the manuscript.

Field-specific reporting

Please select the one below that is the best fit for your research. If you are not sure, read the appropriate sections before making your selection.

☒ Life sciences ☐ Behavioural & social sciences ☐ Ecological, evolutionary & environmental sciences

For a reference copy of the document with all sections, see nature.com/documents/nr-reporting-summary-flat.pdf

Life sciences study design

All studies must disclose on these points even when the disclosure is negative.

Sample size	No statistical method was used to pre-determine the sample size. Sample numbers were based on tissue availability and standard practice in the field for mechanistic studies. Key experiments were repeated independently using at least three biological replicates to ensure reproducibility. The sample sizes used are consistent with similar published studies and sufficient to support the conclusions drawn.
Data exclusions	Data exclusions were applied based on strict quality control criteria. Samples with low RNA quality or signs of cell contamination were excluded from the analysis. No data were excluded for statistical reasons, and all data used in the analysis were obtained from samples that met the established quality control standards. All exclusions were applied uniformly across all experimental conditions.
Replication	Biological replicates were used in key experiments to ensure reproducibility. At least three independent biological replicates were included in each condition to confirm the robustness of the findings. Additionally, technical replicates were performed to account for variability within experimental procedures. The use of multiple replicates ensures the reliability and statistical power of the results.

Randomization	No randomization is applied.
Blinding	Blinding was not applied in this study. The research focused on basic cellular and molecular mechanisms, and no subjective outcome assessments were made that would require blinding. All data were analyzed objectively based on predefined criteria.

Reporting for specific materials, systems and methods

We require information from authors about some types of materials, experimental systems and methods used in many studies. Here, indicate whether each material, system or method listed is relevant to your study. If you are not sure if a list item applies to your research, read the appropriate section before selecting a response.

Materials & experimental systems

n/a	Involved in the study	
☐	☒	Antibodies
☐	☒	Eukaryotic cell lines
☒	☐	Palaeontology and archaeology
☒	☐	Animals and other organisms
☒	☐	Clinical data
☒	☐	Dual use research of concern
☒	☐	Plants

Methods

n/a	Involved in the study	
☒	☐	ChIP-seq
☐	☒	Flow cytometry
☒	☐	MRI-based neuroimaging

Antibodies

Antibodies used

All antibodies were diluted in TBST buffer with 5% non-fat milk for western blot.beta-actin (Santa Cruz, Sc-47778, 1:30000),ZFP42 (Invitrogen, 710190,1:2000), CRY1 (Proteintech, 13474-1-AP,1:3000),Anti-mouse IgG, HRP-linked Antibody (Cell Signaling Technology, 1:2000),Anti-rabbit IgG, HRP-linked Antibody (Cell Signaling Technology, 1:2000),Keratin 1 (Biolegend, 905204, 1:200),MKI67 (Abcam, AB16667, 1:200),Filaggrin (Abcam, ab218397,1:100) ,CoraLite488-conjugated Goat Anti-Rabbit IgG(H+L) (Invitrogen, A11034, 1:200),CoraLite594 – conjugated Goat Anti-Mouse IgG(H+L)(Invitrogen, A21203, 1:200),FITC-conjugated CD49f (BioLegend, 313605,1:20),APC-conjugated CD71 (BioLegend, 334107,1:20), APC-conjugated CD34 (BioLegend, 128611,1:20), KRT14 (Abcam, ab181595,1:200) , keratin 10 (Abcam: ab76318)

Validation

beta-actin (Santa Cruz, Sc-47778, 1:30000),https://www.scbt.com/p/beta-actin-antibody-c4?requestFrom=search;
ZFP42 (Invitrogen, 710190,1:2000),https://www.thermofisher.cn/cn/zh/antibody/product/Rex1-Antibody-clone-14HCLC-Recombinant-Polyclonal/710190;
CRY1 (Proteintech, 13474-1-AP,1:3000),https://www.ptgcn.com/products/CRY1-Antibody-13474-1-AP.htm;
Anti-mouse IgG, HRP-linked Antibody (Cell Signaling Technology, 1:2000),https://www.cellsignal.com/products/secondary-antibodies/anti-mouse-igg-hrp-linked-antibody/7076;
Anti-rabbit IgG, HRP-linked Antibody (Cell Signaling Technology, 1:2000),https://www.cellsignal.com/products/secondary-antibodies/anti-rabbit-igg-hrp-linked-antibody/7074;
Keratin 1 (Biolegend, 905204, 1:200),https://www.biolegend.com/en-us/products/purified-anti-keratin-1-antibody-13412;
MKI67 (Abcam, AB16667, 1:200),https://www.abcam.cn/ki67-antibody-sp6-ab16667.html;
Filaggrin (Abcam, ab218397,1:100) ,https://www.abcam.cn/filaggrin-antibody-flg1562-ab218397.html;
CoraLite488-conjugated Goat Anti-Rabbit IgG(H+L) (Invitrogen, A11034, 1:200),https://www.thermofisher.cn/cn/zh/antibody/product/Goat-anti-Rabbit-IgG-H-L-Highly-Cross-Adsorbed-Secondary-Antibody-Polyclonal/A-11034;
CoraLite594 – conjugated Goat Anti-Mouse IgG(H+L)(Invitrogen, A21203, 1:200),https://www.thermofisher.cn/cn/zh/antibody/product/Donkey-anti-Mouse-IgG-H-L-Highly-Cross-Adsorbed-Secondary-Antibody-Polyclonal/A-21203;
FITC-conjugated CD49f (BioLegend, 313605,1:20),https://www.biolegend.com/en-ie/products/fitc-anti-human-mouse-cd49f-antibody-2606;
APC-conjugated CD71 (BioLegend, 334107,1:20),https://www.biolegend.com/en-ie/products/apc-anti-human-cd71-antibody-7517;
APC-conjugated CD34 (BioLegend, 128611,1:20),https://www.biolegend.com/en-ie/products/apc-anti-mouse-cd34-antibody-6520;
KRT14 (Abcam, ab181595,1:200) ,https://www.abcam.com/en-us/products/primary-antibodies/cytokeratin-14-antibody-epr17350-cytoskeleton-marker-ab181595;
KRT10 (Abcam, ab76318,1:200) ,https://www.abcam.cn/products/primary-antibodies/cytokeratin-10-antibody-ep1607ihcy-cytoskeleton-marker-ab76318;

Eukaryotic cell lines

Policy information about cell lines and Sex and Gender in Research

Cell line source(s)	HEK 293 cells were obtained from ATCC .

Authentication	All cell lines used in this study were authenticated by STR profile report.
Mycoplasma contamination	All cell lines in our laboratory were routinely tested for mycoplasma contamination and cells used in this study were mycoplasma free.
Commonly misidentified lines (See ICLAC register)	No commonly misidentified cell lines were used in this study.

Plants

Seed stocks	*Report on the source of all seed stocks or other plant material used. If applicable, state the seed stock centre and catalogue number. If plant specimens were collected from the field, describe the collection location, date and sampling procedures.*
Novel plant genotypes	*Describe the methods by which all novel plant genotypes were produced. This includes those generated by transgenic approaches, gene editing, chemical/radiation-based mutagenesis and hybridization. For transgenic lines, describe the transformation method, the number of independent lines analyzed and the generation upon which experiments were performed. For gene-edited lines, describe the editor used, the endogenous sequence targeted for editing, the targeting guide RNA sequence (if applicable) and how the editor was applied.*
Authentication	*Describe any authentication procedures for each seed stock used or novel genotype generated. Describe any experiments used to assess the effect of a mutation and, where applicable, how potential secondary effects (e.g. second site T-DNA insertions, mosiacism, off-target gene editing) were examined.*

Flow Cytometry

Plots

Confirm that:

☒ The axis labels state the marker and fluorochrome used (e.g. CD4-FITC).

☒ The axis scales are clearly visible. Include numbers along axes only for bottom left plot of group (a 'group' is an analysis of identical markers).

☒ All plots are contour plots with outliers or pseudocolor plots.

☒ A numerical value for number of cells or percentage (with statistics) is provided.

Methodology

Sample preparation	To determine the cell size, primary human epidermal keratinocytes was seeded on 6 cm dishes at a 80% confluence. Forty-eight hours, cells were harvested and subjected to FACS analysis.
Instrument	All samples were acquired on Beckman Coulter CytoFLEX LX flow cytometer.
Software	FlowJo 10.4.
Cell population abundance	At least 10000 cells were collected for each sample during Flow Cytometry analysis.
Gating strategy	Single-cell suspensions were first gated based on forward scatter (FSC-A) and side scatter (SSC-A) to exclude debris. Doublets were removed by gating on FSC-H versus FSC-A.Live, single cells en analyzed for surface marker expression. Epidermal stem/progenitor populations were identified using CD49f, CD71, CD34d intracellular KRT1, and intracellular KRT14. Gating thresholds were established using fluorescence minus one (FMO) controls and isotype controls to ensure accurate discrimination of positive and negative populations. All flow cytometry data were analyzed using FlowJo software (10.4).

☒ Tick this box to confirm that a figure exemplifying the gating strategy is provided in the Supplementary Information.
